# Recent Progress in Gel Polymer Electrolytes for Lithium-Based Batteries: Design Strategies and Practical Applications

**DOI:** 10.3390/polym18212779

**Published:** 2026-11-07

**Authors:** Md Murshed Bhuyan, Kyungjun Lee

**Affiliations:** Department of Mechanical, Smart, and Industrial Engineering (Mechanical Engineering Major), Gachon University, 1342, Seongnam-daero, Sujeong-gu, Seongnam-si 13120, Republic of Korea; mdmurshed86@gachon.ac.kr

**Keywords:** gel polymer electrolytes, lithium-ion batteries, in situ polymerization, ion transport, high nickel cathodes, thermal safety

## Abstract

Gel polymer electrolytes are attractive quasi-solid electrolyte systems that combine the high ionic mobility and electrode wetting of liquid electrolytes with the dimensional stability and reduced leakage of polymer networks. Their value for high-performance lithium-ion batteries is increasingly determined not only by bulk ionic conductivity, but also by Li^+^ selectivity, solvent retention, mechanical integrity, electrochemical stability, and the ability to form stable interfaces with high-voltage cathodes and practical graphite and Si-containing anodes. This review critically summarizes recent progress in GPEs through a structure, transport, interface-performance framework, with particular emphasis on developments reported in recent years while retaining foundational studies that established the field. Representative polymer hosts, including PVDF/PVDF-HFP, PEO, PAN, PMMA and methacrylate networks, cellulose-derived matrices, and emerging functional polymers, are compared according to their molecular functionality and practical limitations. Major performance-enhancement strategies-including blending, crosslinking, inorganic or nanostructured fillers, functional additives, nonflammable components, self-healing chemistries, and in situ polymerization-are then evaluated in terms of how they regulate ion transport, interphase chemistry, mechanical behavior, thermal safety, and manufacturability. Recent demonstrations in NCM/graphite and NCM/Si graphite pouch cells show that conformal in situ gelation, LiF-rich interphases, adaptive network mechanics, and low-reactivity liquid phases can translate materials-level improvements into high-rate cycling, long life, and improved abuse tolerance. Remaining challenges include conductivity, mechanical strength, high-voltage oxidation and wide-temperature operation. Future GPE development will therefore require integrated molecular, interfacial, and cell-level design rather than optimization of isolated membrane properties.

## 1. Introduction

**Figure 1:**
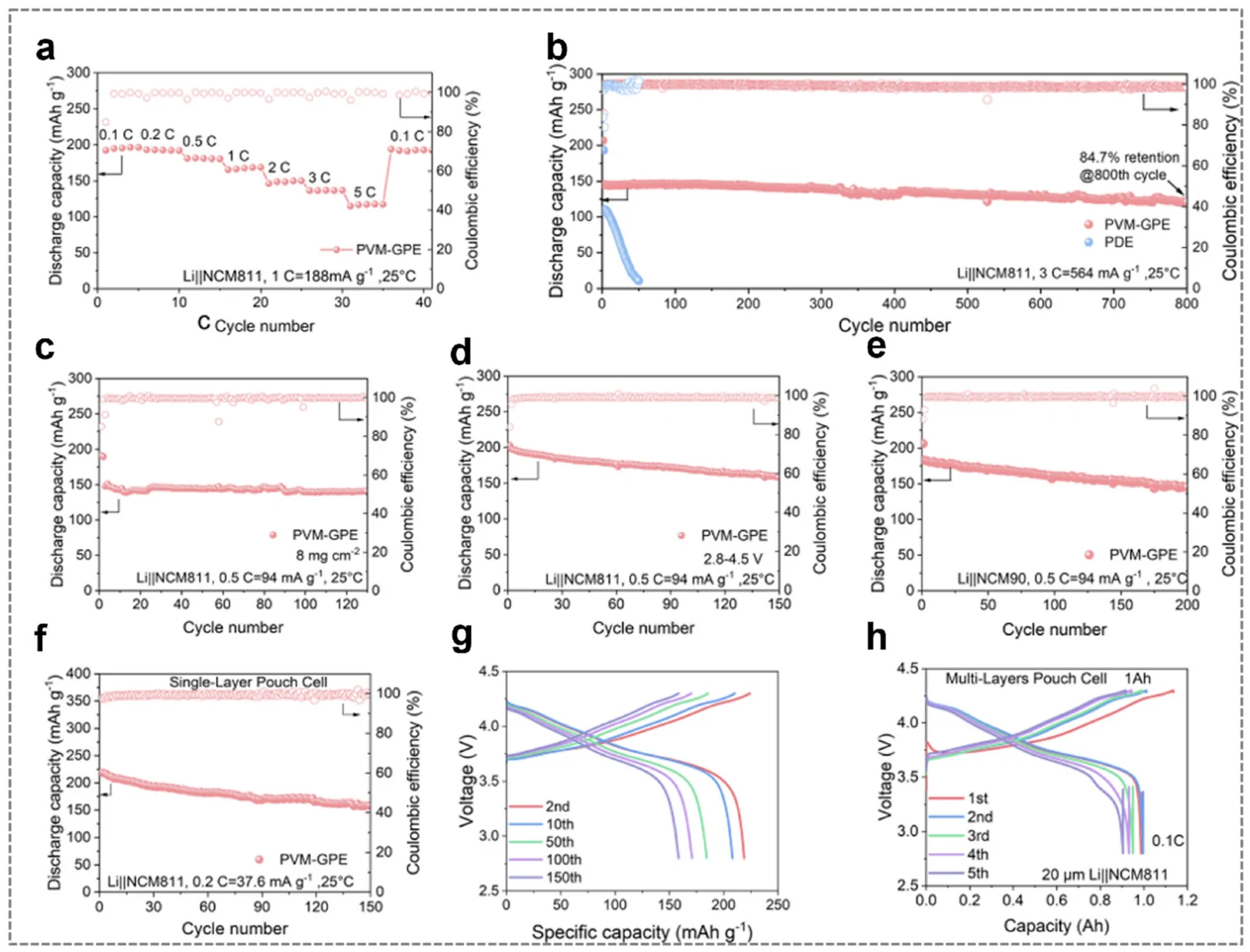
Interphase engineering and polymerization control in gel polymer electrolytes. (**a**–**e**) Li||NCM811 rate performance and cycling at 3 C with fluorine-free PVM-GPE. Cycling with increased NCM811 loading, a 4.5 V cutoff, and NCM90, respectively. (**f**,**g**) Single-layer pouch-cell cycling and voltage profiles. (**h**) Initial five cycles of a multilayer 1 Ah pouch cell. Reproduced from reference [20].

**Figure 2:**
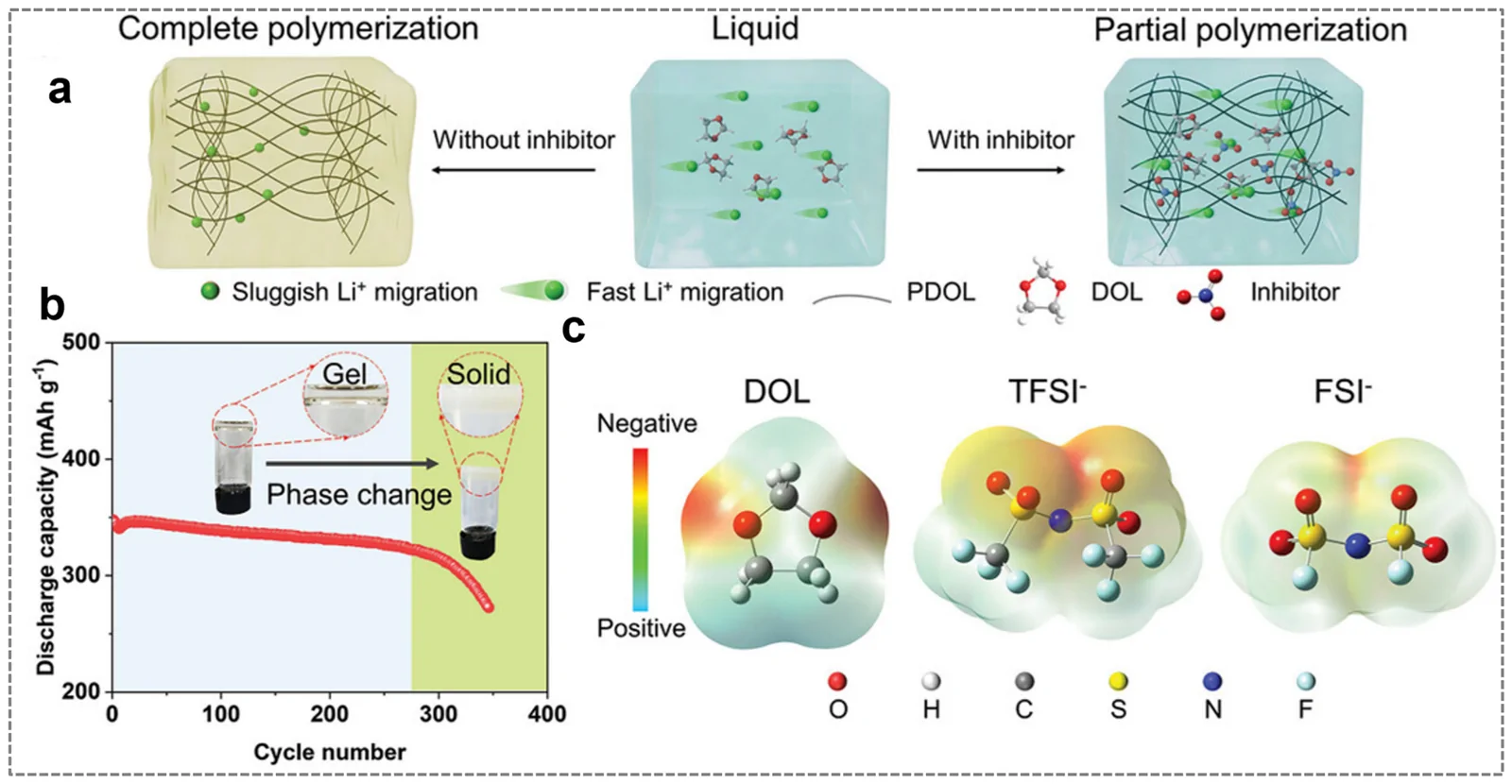
(**a**) Inhibitor-regulated DOL polymerization. (**b**) Graphite||Li half-cell capacity decay associated with uninhibited gel-to-solid evolution; (**c**) Electrostatic-potential maps of DOL, TFSI^−^, and FSI^−^. Reproduced from reference [21].

Lithium-ion batteries (LIBs) underpin portable electronics, electric transportation, and stationary energy storage because they combine high round-trip efficiency, high specific energy, and long cycle life. Continued demand for higher energy density, faster charging, broader operating temperatures, and improved abuse tolerance has placed the electrolyte under increasing scrutiny. Conventional nonaqueous liquid electrolytes remain difficult to replace because they provide high room-temperature ionic conductivity and efficiently wet porous electrodes. However, their volatile and flammable organic solvents can intensify leakage, gas generation, and thermal runaway once electrochemical or thermal instability is triggered [1,2,3,4,5,6]. Fully inorganic solid electrolytes and dry solid polymer electrolytes can suppress leakage and reduce solvent volatility, but they often introduce other limitations, including insufficient room-temperature transport, rigid or imperfect contact with porous electrodes, and processing constraints. GPEs occupy an intermediate regime in which a polymer network immobilizes a liquid electrolyte, plastic crystal, ionic liquid, or other mobile phase. This architecture can preserve liquid-like transport and conformal electrode contact while improving shape retention and reducing free-liquid leakage [7,8,9,10,11,12]. Importantly, however, a GPE should not be considered intrinsically nonflammable; its thermal safety still depends strongly on the chemistry and quantity of the retained liquid phase, the polymer decomposition pathway, and the reactions occurring at charged electrodes. The performance of a GPE is therefore intrinsically multi-objective. Polymer chemistry and network topology determine crystallinity, free volume, solvent affinity, elastic response, and the local coordination environment; the liquid phase governs a large fraction of ionic mobility and interfacial wetting. The salt and additives control solvation and interphase formation, and the electrode-facing region ultimately determines whether stable solid-electrolyte and cathode-electrolyte interphases can be maintained. Increasing solvent content or segmental mobility generally improves conductivity but can reduce modulus and solvent retention, whereas stronger crosslinking improves dimensional stability but may increase transport resistance. Similar trade-offs appear between oxidative stability and wetting, flame resistance and low-temperature transport, and strong adhesion and interfacial impedance [13,14,15,16]. Recent research has shifted from simple solvent-uptake membranes toward chemically engineered gel polymer electrolytes (GPEs) incorporating fluorinated or phosphorus-containing functional groups, ion-selective interactions, adaptive polymer networks, reactive fillers, and in situ polymerization strategies [17,18,19]. Recent GPE developments highlight two complementary design priorities: stabilizing electrode interphases and preserving ion-conducting pathways throughout operation. Fluorine-free interphase engineering provides one example of this approach. Xu et al. [20] developed an in situ copolymerized vinylene carbonate/N,N′-methylenebisacrylamide electrolyte (PVM-GPE) that combines Li^+^ coordinating segments with anion-binding interactions. The resulting Li_2_O-rich, Li_3_N-containing solid-electrolyte interphase was associated with improved interfacial transport and more uniform lithium deposition. Li||NCM811 cells delivered 117.1 mAh g^−1^ at 5 C and retained 84.7% of their initial capacity after 800 cycles at 3 C with a 4.3 V upper cutoff (Figure 1a,b). Separate evaluations examined increased cathode loading, a 4.5 V cutoff, and NCM 90 cathodes (Figure 1c–e). Pouch-cell testing extended the evaluation to 150 cycles in a single-layer configuration and an initial five-cycle demonstration in a multilayer 1 Ah cell (Figure 1f–h). Complementing interphase design, the evolution of the polymer network can determine electrolyte durability. Liu et al. [21] showed that continued polymerization of 1,3-dioxolane (DOL) in a LiFSI-containing electrolyte progressively transformed the gel into a solid polymer electrolyte, accompanied by increased interfacial resistance and accelerated capacity loss in graphite||Li half-cells (Figure 2a,b). Their electrostatic-potential analysis supported a proposed initiation mechanism involving Lewis-acidic sites in FSI^−^ (Figure 2c). Introducing LiNO_3_ moderated this process through preferential interactions between NO_3_^−^ and these sites, limiting further DOL polymerization. The resulting partially polymerized electrolyte, GPELN, maintained an amorphous gel structure and an ionic conductivity of 5.10 mS cm^−1^ after 30 days of aging. Importantly, Liu et al. also demonstrated this strategy in LFP||graphite pouch full cells, achieving 80.02% capacity retention after 715 cycles at 5 C charging and 1 C discharging [21]. Together, these studies illustrate why GPE development must address both interphase chemistry and the persistence of the conductive gel state. Whereas the fluorine-free PVM-GPE demonstrates interphase-directed performance improvements in lithium-metal cells, inhibitor-regulated polymerization provides complementary evidence in graphite-based LIBs, connecting molecular-level electrolyte design with fast-charging full-cell operation.

The most consequential advance for LIB relevance is the increasing use of graphite, Si-containing anodes, high-Ni NCM cathodes, and Ah-scale pouch cells rather than relying exclusively on Li-metal half-cells. Accordingly, this review emphasizes the structure-transport-interface performance relationship of GPEs for practical LIBs. Foundational literature is retained where it defines polymer chemistry or transport concepts, while recent years’ studies are prioritized when discussing high-voltage operation, wide-temperature behavior, thermal safety, in situ processing, and full-cell validation. Li-metal cells are included when they isolate an electrolyte or interfacial mechanism, but their results are explicitly distinguished from evidence obtained in conventional LIB architectures.

**Figure 3:**
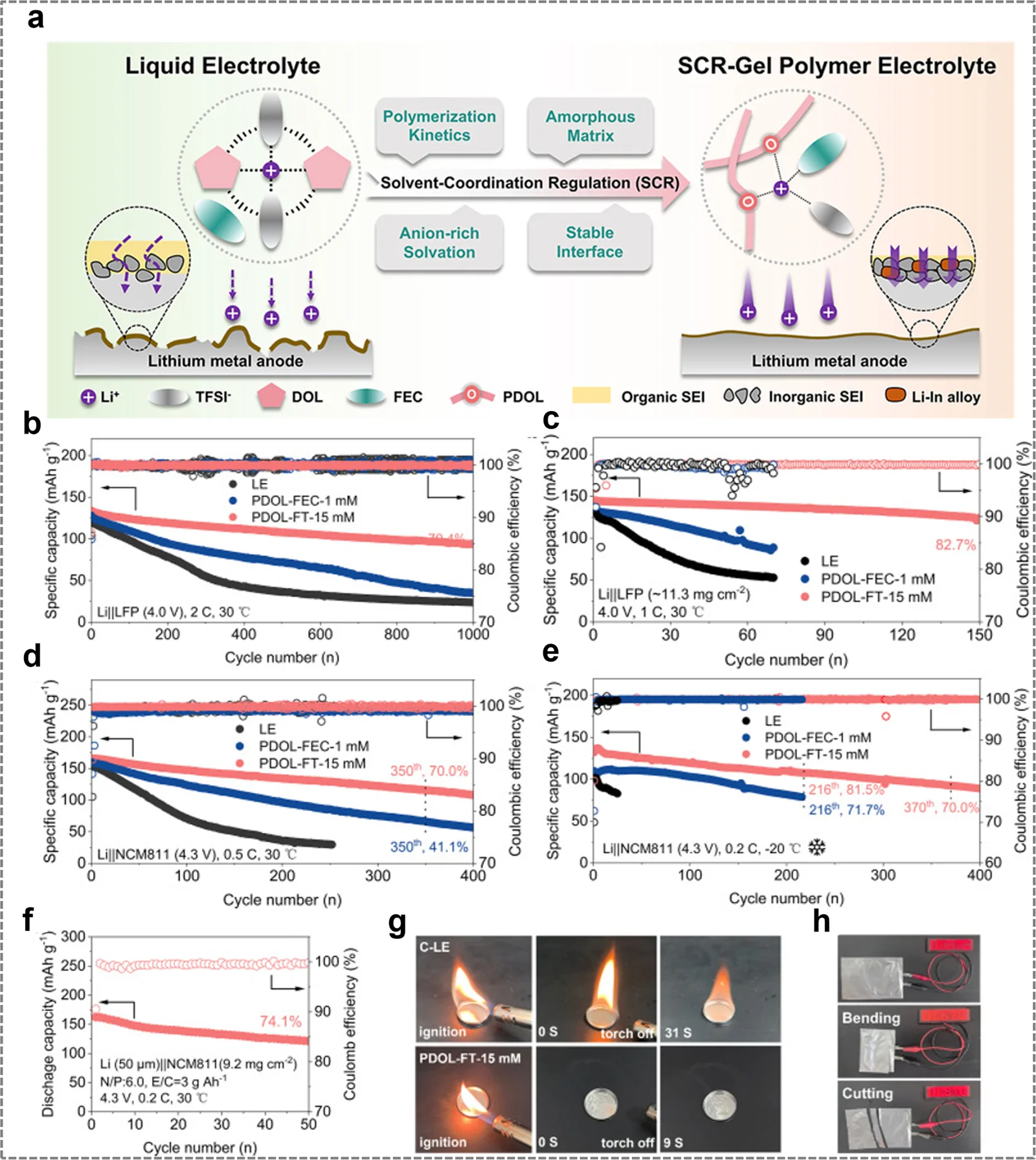
Solvent-coordination-regulated gel polymer electrolyte. (**a**) Design concept integrating polymerization, solvation, and interphase regulation. (**b**,**c**) Li||LFP cycling performance. (**d**,**e**) Li||NCM811 cycling at 30 and −20 °C, respectively. (**f**) Li||NCM811 cycling with a 50 μm lithium anode and an electrolyte loading of 3 g Ah^−1^. (**g**) Flame-retardancy comparison of C-LE and PDOL-FT-15 mM electrolytes (0 s” indicates the moment of torch removal, with subsequent times showing the elapsed duration). (**h**) Pouch-cell LED operation during bending and partial cutting of the packaging. Reproduced from reference [22].

The development of high-performance GPEs requires more than the physical immobilization of a liquid phase, as polymer formation, ion solvation, and interphase chemistry must be designed as coupled processes. This principle is illustrated by the solvent-coordination-regulation (SCR) strategy reported by Chen et al. [22], which connects the polymerization of 1,3-dioxolane (DOL) with the subsequent electrochemical function of the resulting gel (Figure 3a). Preferential coordination of triethyl phosphate to In^3+^ moderates the effective Lewis acidity of In(OTf)_3_, enabling controlled ring-opening polymerization at increased initiator loading, while fluoroethylene carbonate helps preserve an amorphous poly(DOL) matrix. Concurrently, polymerization reorganizes the Li^+^ coordination environment toward an anion-rich solvation structure, facilitating desolvation and favoring inorganic interphase formation. Together with indium-derived alloy formation, these changes produce a Li-In/LiF-rich solid-electrolyte interphase that promotes more uniform lithium deposition and improved interfacial transport. The significance of this approach therefore lies in integrating processing control with solvation and interphase engineering, rather than treating gelation solely as a means of retaining liquid electrolyte. The electrochemical and functional demonstrations in Figure 3b–h illustrate the consequences of this integrated design. With an upper cutoff voltage of 4.3 V, Li||NCM811 cells retained approximately 70% of their initial capacity after 350 cycles at 0.5 C and 30 °C, and after 370 cycles at 0.2 C and 20 °C. The optimized formulation also exhibited reduced flammability, while the corresponding pouch cell maintained LED operation during bending and partial cutting of the outer packaging [22]. Although these results were established in lithium-metal cells rather than conventional lithium-ion full cells, they provide a mechanistic example of how polymerization control, solvation regulation, and interphase design can be combined within a GPE. For LIB-focused development, the corresponding challenge is to translate these principles into formulations compatible with graphite and silicon-containing anodes under realistic full-cell conditions.

## 2. Fundamentals of Gel Polymer Electrolytes

### 2.1. Composition and Classification

**Figure 4:**
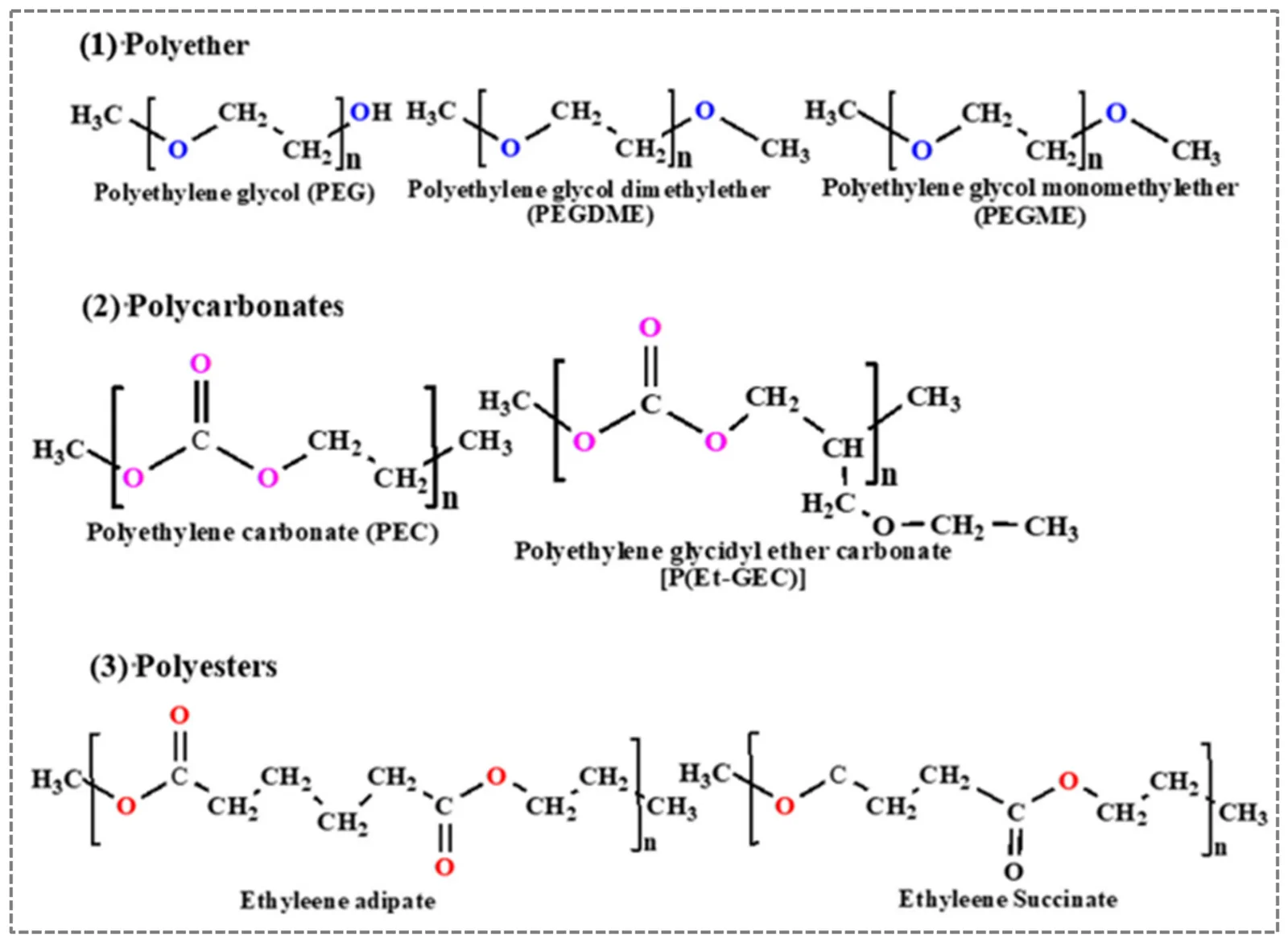
Representative oxygen-containing polymer structures for polymer electrolyte design. (**1**) Polyethers: PEG, PEGDME, and PEGME. (**2**) Polycarbonates: PEC and P(Et-GEC). (**3**) Adipate- and succinate-based polyesters. The structures highlight the ether, carbonate, and ester functionalities of the respective families. Reproduced from reference [25].

A GPE generally contains four functional components: a polymer host, a lithium salt, a liquid or plasticizing phase, and optional fillers or functional additives. The polymer network provides dimensional continuity and controls porosity, swelling, solvent retention, and part of the local coordination environment; the mobile phase provides most of the liquid-like ion-conduction pathway; the lithium salt supplies charge carriers; and additives can modify flammability, solvation, interphase chemistry, or mechanical behavior [6,10,16,23]. GPEs are commonly classified by how the polymer network is formed. Physical gels rely on chain entanglement, crystallites, pores, or noncovalent interactions and are often simple to process, but they can be more susceptible to solvent migration or mechanical softening. Chemically crosslinked gels contain permanent or dynamic covalent junctions that improve dimensional stability, although excessive network density can suppress solvent uptake and segmental mobility. A second practically important distinction is between preformed ex situ membranes and in situ formed GPEs. In the latter, a low-viscosity precursor infiltrates the porous electrodes and is subsequently polymerized inside the assembled cell, improving interfacial conformity and reducing voids [24]. Modern high-performance systems increasingly combine physical confinement, chemical crosslinking, and interphase-forming chemistry rather than relying on a single gelation mechanism. Polymer backbone chemistry provides the molecular basis for lithium-salt solvation and the structural organization of polymer electrolytes. As illustrated in Figure 4, representative oxygen-containing families include polyethers, polycarbonates, and polyesters [25]. Polyethers, including polyethylene glycol (PEG) and its methyl ether derivatives, contain repeating ether-oxygen sites that can coordinate Li^+^. The combination of electron-donating oxygen atoms and flexible ethylene oxide segments underpins the use of these chains as solvating components in polymer electrolytes. Polycarbonates, represented by polyethylene carbonate (PEC) and P(Et-GEC), introduce carbonate linkages, whereas the adipate- and succinate-based polyesters contain ester groups separated by aliphatic segments. These structures illustrate distinct arrangements of oxygen-containing functionalities within polymer chains, providing a chemical framework for discussing polymer ion interactions rather than a direct comparison of electrolyte performance. Although these backbones are introduced in the source review in the context of solid polymer electrolytes, their relevance to GPE design must be considered together with the retained liquid phase. In GPEs, the polymer framework confines the liquid electrolyte, while its composition and network structure influence electrolyte retention, mechanical integrity, and electrode contact [25]. Importantly, an oxygen-containing polymer does not necessarily need to function as the primary structural matrix. For example, the source review describes the incorporation of PEGDME as a plasticizer within a PVDF-HFP framework, demonstrating that solvation and structural support can be provided by different components. Therefore, polymer backbone selection should be considered alongside electrolyte formulation and network design, as the chemical structure of an individual polymer alone cannot fully determine the performance of the resulting gel electrolyte.

The membrane photographs and porous-network morphology illustrate the combination of structural continuity, electrolyte accommodation, and flexibility after swelling. Increasing the PEG content from 2.5% to 20% increased the tensile strength of the CP precursor membranes from approximately 34 to 211 MPa, which the authors attributed to hydrogen bonding between cellulose and PEG. However, the highest mechanical strength did not coincide with the most favorable transport properties: the electrolyte containing 5% PEG, GCP-2, exhibited the maximum room-temperature ionic conductivity of 3.31 mS cm^−1^ and a reported Li^+^ transference number of 0.63. Further PEG addition reduced electrolyte uptake and conductivity, reflecting the competing effects of network densification, cellulose anion interactions, and ether-group mobility. The authors proposed that PEG-assisted Li^+^ coordination and dissociation facilitate transport at moderate PEG contents, whereas stronger interchain interactions at higher contents increasingly constrain this process. Li||NCM523 cells incorporating GCP-2 delivered an initial discharge capacity of 159.3 mAh g^−1^ at 0.2 C, although the charge–discharge profiles showed increasing polarization at higher rates [26].

Acetylated nanofibrils were processed into films by solvent casting and subsequently activated by liquid-electrolyte immersion. Replacing hydroxyl groups with ester groups improved carbonate-electrolyte affinity and reduced water affinity, while sufficiently extensive acetylation decreased crystalline ordering. At a degree of substitution of 2.37, the resulting GACNF-3 electrolyte achieved an electrolyte uptake of 301%, an ionic conductivity of 2.73 mS cm^−1^, and a Li^+^ transference number of 0.65. These transport improvements were accompanied by a mechanical trade-off: disruption of interfibrillar hydrogen bonding reduced the precursor-film tensile strength from 99.51 to 37.66 MPa, while the elongation at break reached 10.55%. The corresponding Li||LiFePO_4_ cell delivered an initial discharge capacity of 153 mAh g^−1^ and retained 88.8% of its capacity after 100 cycles at 0.2 C. The authors associated this behavior with improved transport and interfacial compatibility, together with reduced moisture- and hydroxyl-associated side reactions, as represented in the durability schematic [27].

### 2.2. Ion Transport and the Role of the Polymer Matrix

Li-ion transport in GPEs cannot be described by a single polymer-hopping mechanism. In many formulations, a substantial fraction of ionic motion occurs through the absorbed liquid phase, while the polymer modifies salt dissociation, solvent organization, tortuosity, and ion motion near polymer-rich or filler-rich domains. Polar groups such as ether oxygen, carbonyl, nitrile, hydroxyl, and fluorinated functionalities influence Li^+^ coordination and anion association. In PEO-containing systems, Li^+^ can coordinate to ether oxygens and exchange between neighboring sites through segmental motion, so crystallinity strongly suppresses transport and is commonly reduced by copolymerization, blending, plasticization, or filler addition [15,28,29]. In PVDF/PVDF-HFP matrices, the polar fluorinated backbone and reduced crystallinity of HFP-containing copolymers favor electrolyte uptake and a dielectric environment that can support salt dissociation, although conduction is still often dominated by the liquid-rich phase. PAN provides strongly polar nitrile groups, PMMA and related methacrylates provide carbonyl-rich and readily crosslinkable frameworks, and cellulose-derived hosts supply hydroxyl-rich networks that are mechanically robust but require strict moisture control [17,18,19,20,30,31,32,33,34,35,36,37,38].

**Figure 5:**
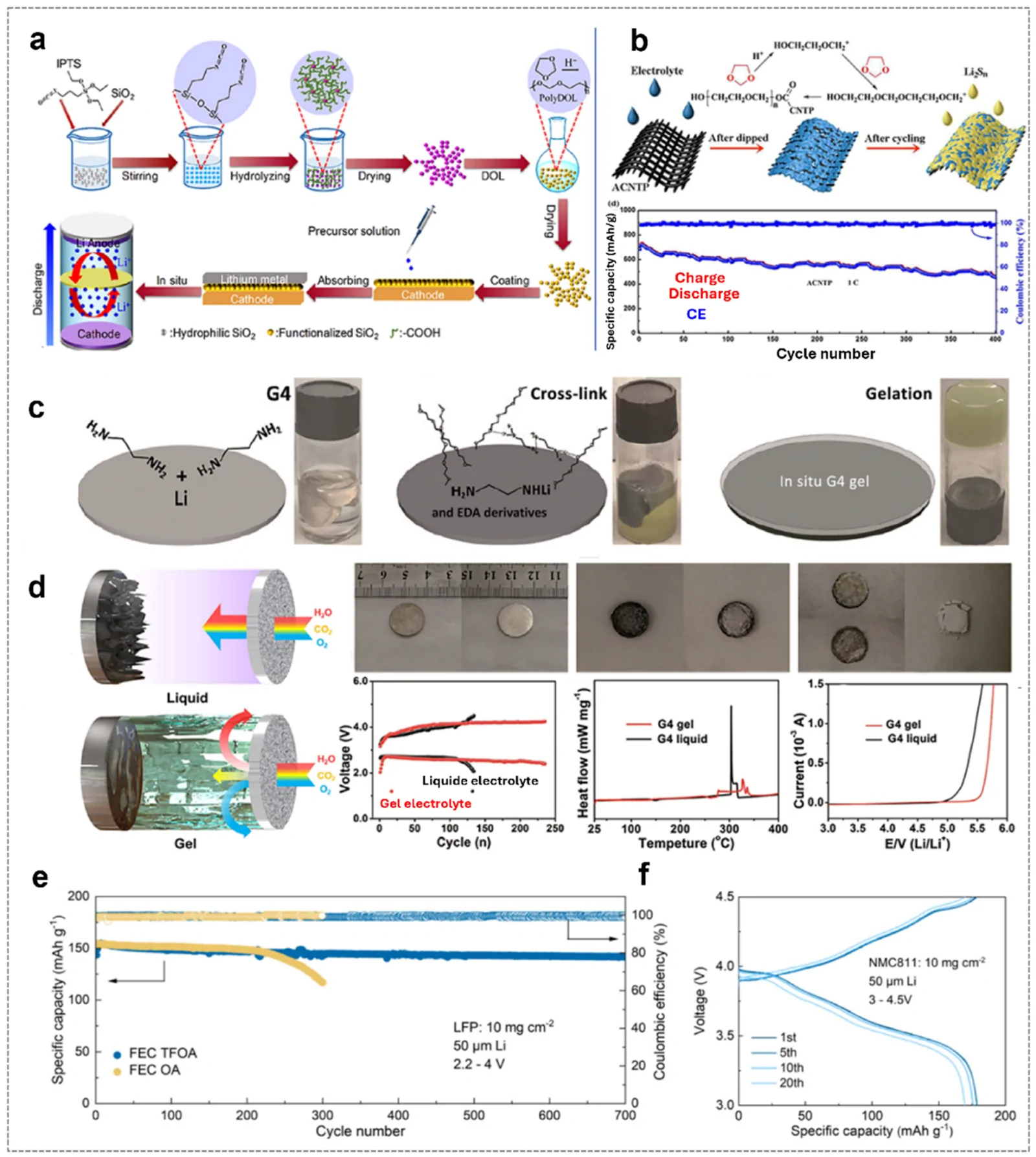
Polymer-matrix regulation of ion transport and interfacial stability in gel electrolytes. (**a**) Preparation of a functionalized SiO2-supported GPE. (**b**) Localized DOL polymerization on acidized carbon nanotube paper and the corresponding Li-S cycling performance. (**c**) Li- O-mediated gelation of G4 using EDA-derived lithium species. (**d**) Protective function, cycling behavior, thermal response, and oxidative stability of the G4 gel in lithium-air systems (adapted from Reference [41], open access). (**e**) Li||LFP cycling with FEC-TFOA and FEC-OA gels at 0.5 C. (**f**) Li||NCM811 voltage profiles with FEC-TFOA at 0.2 C and a 4.5 V upper cutoff (adapted from reference [42]).

High total ionic conductivity alone is therefore an incomplete descriptor of GPE transport. The Li-ion transference number, ion-pairing state, activation behavior with temperature, tortuosity, membrane thickness, and interfacial resistance should be considered together. Under high current density, mobile anions can generate concentration gradients and polarization even when bulk conductivity is high. Strategies such as anion-binding functional groups, immobilized anionic moieties, Lewis-acidic fillers, or specifically designed hydrogen-bonding sites can increase the relative contribution of Li^+^ transport [39]. Recent high-current-density GPEs illustrate this principle by combining rapid Li^+^ transport channels with anion-anchoring groups rather than pursuing conductivity alone [40]. For meaningful comparison, reported conductivity and transference values should be accompanied by the measurement temperature, membrane thickness, liquid fraction, salt concentration, and test method. The polymer matrix influences GPE performance through both molecular coordination and the spatial organization of the conducting phase. Beyond retaining liquid electrolyte, an appropriately designed network can preserve electrode contact, regulate the passage of dissolved species, and modify interfacial reactions. The examples are summarized by Ma et al. [41]. illustrate these complementary functions. In silica-supported architecture, surface-functionalized SiO_2_ promotes DOL gelation, followed by in situ polymerization of tripropylene glycol diacrylate to form a reinforced electrolyte with reduced electrode-electrolyte interfacial resistance (Figure 5a). In contrast, acidized carbon nanotube paper induces localized DOL polymerization, producing a thin, ion-selective layer that restricts polysulfide migration while allowing Li^+^ transport in Li-S cells (Figure 5b). Importantly, the review notes that this gel exhibits lower Li^+^ diffusivity than its liquid counterpart; its effectiveness therefore depends on maintaining a sufficiently thin layer to limit the transport penalty. This example distinguishes selective species confinement from an intrinsic increase in bulk ion mobility [41]. Network formation can also couple electrolyte immobilization with protection of the electrode surface. In the tetraethylene glycol dimethyl ether (G4) system, the reaction of ethylenediamine (EDA) with lithium produces LiEDA species that initiate Li-O-mediated crosslinking and progressive gelation (Figure 5c). As described in [41], the resulting gel suppresses H_2_O and CO_2_ penetration, limits lithium corrosion, and improves cycling stability in ambient lithium air batteries. The accompanying permeability, cycling, thermal, and oxidative-stability measurements illustrate this protective function (Figure 5d). Here, the matrix contributes to sustained electrochemical operation by controlling the electrode’s chemical environment, rather than solely by modifying the mobility of the charge carriers. At the molecular level, Li et al. [42] demonstrated that ion transport depends on the relative Li^+^ solvating abilities of the polymer and solvent. Comparing networks derived from fluorinated 1H, 1H, 2H, 2H-tridecafluoro-*n*-octyl acrylate (TFOA) and non-fluorinated *n*-octyl acrylate (OA), the authors associated fluorination with weaker Li^+^ polymer interactions. When combined with fluoroethylene carbonate (FEC), the TFOA-based network promoted partial Li^+^ detachment, shorter coordination contact times, and increased participation of anions in the solvation environment. These changes were interpreted as facilitating exchange between neighboring coordination sites. Conversely, strongly solvating solvents such as dimethoxyethane produced nearly complete Li^+^ polymer detachment and micellar structures that impeded transport. Within the investigated formulations, efficient transport therefore required balanced polymer–solvent coordination rather than indiscriminate weakening of Li^+^ binding [42]. The distinction between total conductivity and Li^+^ transport is particularly instructive. The FEC-TFOA gel exhibited slightly lower room-temperature ionic conductivity than FEC-OA, 0.29 versus 0.32 mS cm^−1^, but a higher Li^+^ transference number, 0.55 versus 0.44. The simulations likewise yielded lower individual self-diffusion coefficients for FEC-TFOA despite its higher calculated transference number. Thus, improved Li^+^ selectivity should not be equated with a universal increase in diffusivity. The FEC-TFOA (Figure 5e,f) formulation also favored an inorganic-rich SEI containing LiF and Li_2_O, linking coordination regulation to interfacial stabilization [42].

### 2.3. Key Performance Requirements and Characterization

An effective GPE must simultaneously provide sufficient ion transport, mechanical integrity, electrolyte retention, electrode compatibility, and thermal and electrochemical stability. Electrochemical impedance spectroscopy is commonly used to determine bulk conductivity and follow interfacial resistance. Polarization methods are used to estimate Li^+^ transference, and tensile or dynamic-mechanical measurements probe network robustness. Solvent uptake, swelling, gel fraction, thermal analysis, and flammability tests are essential because the retained liquid phase can dominate both transport and safety. Linear or cyclic voltammetry provides a useful screening measure of apparent electrochemical stability, but an onset potential measured on an inert current collector should not be treated as proof of long-term compatibility with a high-voltage cathode. Voltage-hold experiments, impedance evolution, gas analysis, post-mortem interphase characterization, and prolonged full-cell cycling provide more rigorous evidence [16,43,44,45,46,47,48]. For claims of practical LIB performance, cell configuration, areal loading, N/P ratio, electrolyte quantity, formation protocol, temperature, pressure, voltage window, and pouch- or coin-cell format should be reported alongside conductivity and mechanical properties. Table 1 presents the polymeric matrices with their own advantages and limitations. Reporting discipline is particularly important because seemingly similar GPEs can differ substantially in liquid content, porosity, and test severity.

## 3. Polymer Matrices for High-Performance GPEs

### 3.1. PVDF- and PVDF-HFP-Based Systems

PVDF and PVDF-HFP remain among the most widely investigated GPE hosts because they combine strong film formation, chemical resistance, and compatibility with carbonate-based electrolytes. PVDF is relatively crystalline, whereas incorporation of HFP disrupts chain packing, increases amorphous character and free volume, and generally improves electrolyte uptake. Consequently, PVDF-HFP is often used as a porous or cross-linkable scaffold when high solvent retention and broad electrochemical compatibility are required [30,49,50]. Its main role should nevertheless be viewed as structural and interfacial rather than as an intrinsically fast Li-ion conductor. Early PVDF-HFP systems demonstrated that ionic liquids can be immobilized within the fluoropolymer network without completely sacrificing electrochemical performance. A PVDF-HFP GPE containing 1-butyl-4-methylpyridinium bis(trifluoromethanesulfonyl)imide was reported to exhibit an electrochemical stability range of approximately 5.5 V versus Li^+^/Li at 20 °C and to support LiFePO_4_-based cells with discharge capacities on the order of 160 mAh g^−1^ [30]. The result illustrates a recurring design principle in fluorinated GPEs: the polymer provides shape and chemical stability, whereas the confined liquid or ionic-liquid phase supplies much of the ionic conductivity. Layered or composite architectures can further decouple mechanical and transport functions. A PVDF/HEC/PVDF sandwich membrane prepared by electrospinning combined a mechanically robust fluoropolymer framework with hydroxyl-rich hydroxyethyl cellulose. In Li/LiFePO_4_ cells, the composite GPE maintained approximately 140 mAh g^−1^ over about 140 cycles at 0.2 C. The authors attributed improved Li-ion transport in part to interactions that restrict PF_6_ motion within the polymer environment [40]. Similarly, porous PVDF-HFP/PEO/SiO_2_ membranes prepared by immersion precipitation achieved strong electrolyte uptake and delivered about 147.7 mAh g^−1^ after 200 cycles in LiFePO_4_ cells [50]. Fluoropolymer GPEs are also attractive platforms for safety-oriented design. A PVDF-HFP matrix plasticized with polyethylene glycol dimethyl ether was prepared by one-pot solvent casting and showed nonflammability, thermal stability approaching 250 °C, and about 98% capacity retention after 60 cycles in the reported cell configuration [51]. In another approach, a PEG-UPy copolymer was incorporated into a PVDF-HFP/PEO matrix to introduce quadruple hydrogen-bonding motifs. The resulting network exhibited room-temperature self-healing and delivered approximately 163 mAh g^−1^ at 1 C in LiFePO_4_-based cells, with stable cycling over 200 cycles [24]. These examples show how PVDF-HFP can serve as a chemically robust base matrix while secondary polymers or supramolecular units provide targeted functions. The broad formulation space of PVDF-based GPEs is both an advantage and a source of ambiguity. Porosity, solvent fraction, HFP content, salt chemistry, support layers, filler dispersion, and polymerization route can influence performance as strongly as the fluoropolymer itself. The most convincing PVDF/PVDF-HFP designs therefore use the fluorinated network as a chemically robust scaffold while deliberately engineering liquid-rich transport pathways and electrode-facing chemistry. This distinction is important when comparing nominally similar PVDF-HFP membranes across studies. Fluorinated polymer hosts, particularly PVDF and PVDF-HFP, remain among the most widely investigated matrices for gel polymer electrolytes because they combine strong electrochemical stability with high polarity, good electrolyte affinity, and intrinsically improved flame resistance. A fluorinated electrospun interlayer on Cu promotes atomic alignment and facilitates gelation-induced formation of an artificial SEI, which in turn enables more uniform Li deposition compared with a conventional gel configuration that leads to uneven lithium growth. This interfacial regulation is directly reflected in the electrochemical results of Cu||NMC cells, where the Cu@GPE configuration shows the most stable cycling behavior and the highest retained areal capacity, whereas the Celgard-based cell fails rapidly and the conventional Cu|cGPE|NMC system shows intermediate behavior. These results suggest that, in PVDF-based systems, the matrix architecture and its interaction with the current collector are as important as bulk ionic transport in determining practical reversibility [52]. A second advantage of fluoropolymer matrices is their contribution to electrolyte safety. A PVDF-HFP-based membrane exhibits strong resistance to ignition and does not sustain vigorous burning during direct flame exposure. This behavior is particularly relevant for GPE design, because one of the major motivations for replacing conventional liquid electrolytes is to reduce flammability while preserving sufficient ionic conductivity and interfacial contact. Taken together, these examples demonstrate that PVDF- and PVDF-HFP-based GPEs offer a versatile platform in which the polymer matrix can simultaneously support electrolyte uptake, stabilize Li deposition, and improve thermal safety. Accordingly, the performance of fluorinated GPEs should be evaluated not only in terms of conductivity, but also in terms of their ability to construct robust interphases and suppress hazardous thermal behavior [53].

### 3.2. PEO-Based GPEs

**Figure 6:**
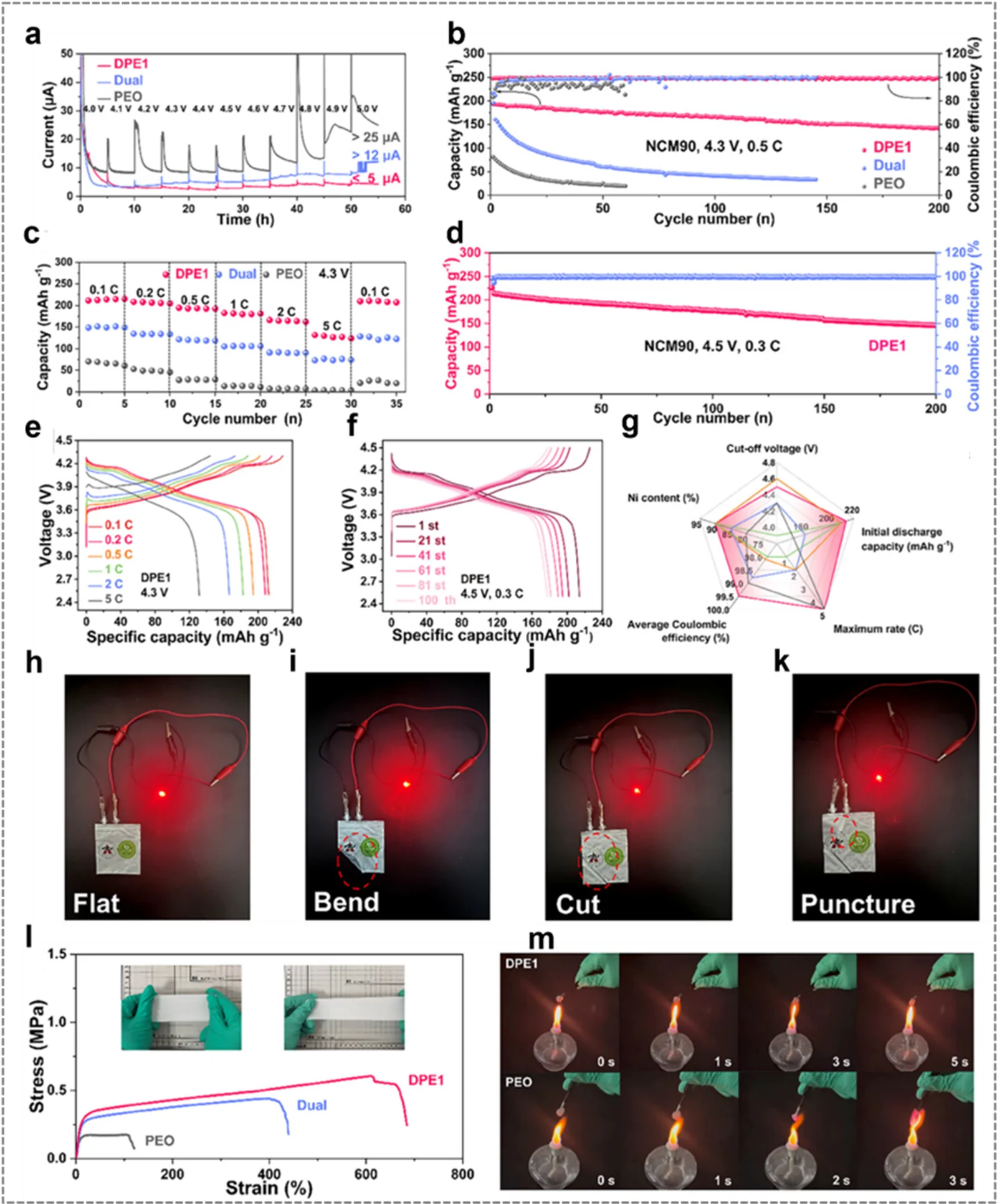
Performance of the PEO-based DPE1 composite electrolyte. (**a**) Electrochemical floating tests at 25 °C. (**b**,**c**) Li||NCM90 cycling and rate performance at a 4.3 V cutoff. (**d**) Cycling at 4.5 V. (**e**,**f**) Rate- and cycle-dependent voltage profiles. (**g**) Literature comparison. (**h**–**k**) Pouch-cell LED operation under flat, bent, cut, and punctured conditions. (**l**) Stress–strain curves. (**m**) Flame-exposure tests. Charge–discharge measurements in (**b**–**f**) were conducted at 60 °C. Reproduced with permission from reference [55].

PEO is a classical polymer-electrolyte host because ether oxygens coordinate Li^+^ and its flexible backbone can support segmental-motion-assisted transport. In dry solid polymer electrolytes, room-temperature crystallinity and limited high-voltage stability are major constraints. In GPEs, however, absorbed solvent, copolymerization, and plasticizing components can strongly suppress crystalline order and shift a larger fraction of transport into liquid-rich domains [15,28,29]. PEO is therefore particularly useful as a soft transport-promoting component in blends or crosslinked networks, although PEO-rich formulations require careful protection against oxidative degradation at high-voltage cathodes. A representative PEO-based GPE demonstrated good ionic transport, thermal stability, and electrochemical cycling in LiFePO_4_ cells while retaining the liquid electrolyte inside a solidified polymer framework [29]. PEO-containing composite systems also illustrate how soft chain segments can complement more rigid structural components. For example, combining PEO with PVDF-HFP and inorganic particles can improve porosity, electrolyte uptake, and mechanical stability simultaneously [50]. The main design constraint is that excessive PEO content can reduce high-voltage oxidative stability, so PEO-rich GPEs are often more attractive when the cathode potential is moderate or when interfacial protection is provided by additives or fluorinated components [15,44,45]. Succinonitrile-containing systems provide another route to plasticize polymer-rich matrices and maintain transport over a wider temperature range. A robust succinonitrile-based gel polymer electrolyte was reported to survive repeated folding and to provide stable cycling at elevated temperature, while flame testing indicated lower flammability than a conventional carbonate-containing gel formulation [54]. Such results reinforce the concept that the transport phase in a GPE need not be restricted to conventional carbonate solvents; plastic crystals and other low-volatility components can be incorporated when thermal or mechanical requirements justify the additional formulation complexity. Wang et al. developed a PEO-based composite electrolyte, DPE1, through an enthalpy–entropy decoupled coordination strategy [55]. Incorporating a poly(ionic liquid) network and LiTFSI/LiDFOB dual salts reduced PEO crystallinity, weakened Li+-based PEO coordination, and promoted a LiF/BF_3_ rich cathode interphase, linking improved ion transport with high-voltage stability. DPE1 achieved an ionic conductivity of 0.117 mS cm^−1^ at 25 °C (Figure 6a–c) and a Li^+^ transference number of 0.71 at 30 °C. At 60 °C, Li||NCM90 cells retained 145.7 mAh g^−1^ after 200 cycles at 0.3 C with a 4.5 V upper cutoff (Figure 6d–g). Improved stretchability, resistance to ignition during 5 s of flame exposure, and continued pouch-cell LED operation after mechanical damage further demonstrated the multifunctional matrix design (Figure 6h–m) [55]. Although DPE1 is classified as a solid composite electrolyte rather than a conventional GPE, it is included here as a related polymer electrolyte system to illustrate transferable principles of polymer coordination, interfacial stabilization, and mechanical reinforcement.

### 3.3. PAN-, PMMA-, and Methacrylate-Based Networks

PAN is attractive because the nitrile group is highly polar and the polymer can form mechanically continuous membranes with good affinity for many liquid electrolytes. PAN is also readily combined with other polymers or inorganic components. Composite PAN/PVA systems and PAN-supported crosslinked GPEs demonstrate how nitrile-containing networks can be used to balance conductivity, mechanical support, and thermal behavior [18,25]. A PE-supported poly(acrylonitrile-co-methyl methacrylate) electrolyte, for example, showed that changing the AN:MMA ratio directly altered electrolyte performance; the optimized composition combined room-temperature conductivity with thermal stability reported up to approximately 270 °C [36]. PMMA and related methacrylate chemistries offer especially broad synthetic flexibility. Carbonyl groups interact favorably with polar solvents, and the monomer chemistry is well suited to free-radical or photo-initiated crosslinking. A poly(methacrylamide) derivative containing terminal nitrile groups was reported to form transparent and flexible gels with oxidation stability to roughly 4.6 V versus Li and glass-transition temperatures below room temperature [35]. Oligo (ethylene glycol) methyl ether methacrylate combined with benzyl methacrylate produced a gel-forming polymer capable of absorbing liquid electrolyte at several times its own mass while retaining sufficient mechanical integrity for separator-like use [37].

Methacrylate chemistry is especially attractive for in situ GPEs because network architecture and functional groups can be adjusted independently. OEGMA-rich units provide flexible, electrolyte-affine domains, while multifunctional acrylates or methacrylates provide permanent crosslinks. More recent systems extend this concept by incorporating fluorinated segments, hydrogen-bonding motifs, or anion-binding groups that simultaneously tune ion transport and interfacial chemistry [38]. This synthetic versatility is a major reason that methacrylate and acrylate networks appear frequently in practical full-cell and pouch-cell demonstrations. A useful development is the deliberate design of adhesive or mechanically adaptive GPEs. Poly(4-hydroxybutyl acrylate)-based gels, for example, demonstrate that electrode adhesion can be treated as a controllable electrolyte property rather than as a secondary consequence of softness [56]. In Si-containing LIBs, dynamic multiple hydrogen bonds have recently been used to maintain contact with expanding SiO_x_/graphite electrodes and enable pressure-free Ah-scale pouch-cell cycling [57]. These results shift the design target from maximizing bulk modulus toward achieving a network that is strong enough to retain electrolyte yet compliant and adhesive enough to follow electrode-level volume changes.

### 3.4. Cellulose and Other Sustainable Polymer Hosts

**Figure 7:**
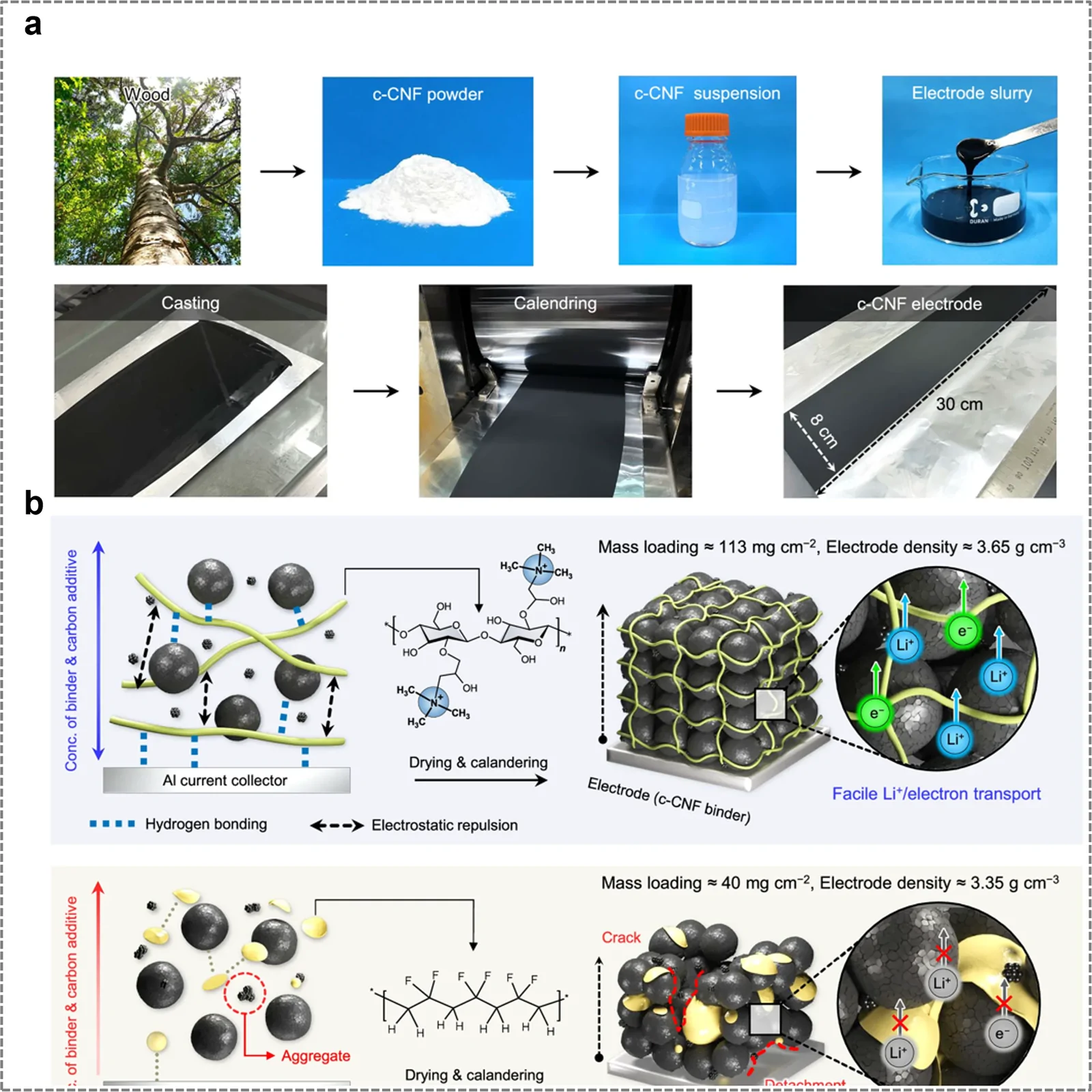
Charge-engineered cellulose nanofibril binders for high-loading NCM811 electrodes: (**a**) Electrode fabrication using wood-derived c-CNF, including suspension preparation, slurry casting, and calendaring; (**b**) Proposed effects of electrostatic repulsion, hydrogen bonding, and nanofibrillar networking on electrode uniformity and transport, compared with PVDF-based electrodes. Reproduced from reference [58].

Cellulose-based GPEs introduce a different design priority: combining electrochemical function with renewable and potentially biodegradable polymer frameworks. Cellulose provides a mechanically strong polysaccharide backbone with abundant hydroxyl groups that can be crosslinked or derivatized. These functional groups also support strong interactions with polar liquid electrolytes, enabling substantial uptake when the membrane microstructure is appropriately controlled [31,32,33,34]. A crosslinked cellulose gel membrane reported by Du and co-workers combined a tensile strength of about 14.6 MPa with electrolyte uptake around 540%; Li/LiFePO_4_ cells delivered approximately 145 mAh g^−1^ in the first cycle at 0.2 C and retained about 90% of capacity after 50 cycles [31]. Introducing polyethylene glycol into a crosslinked cellulose network increased flexibility while maintaining high strength. At an optimized PEG content, Li/GPE/NCM523 cells delivered an initial discharge capacity of roughly 159 mAh g^−1^ at 0.2 C [26]. Acetylated cellulose nanofibers have likewise been used to form GPE films with high uptake, good thermal stability, and LiFePO_4_ cycling retention approaching 89% after 100 cycles at 0.2 C [27]. The value of cellulose is therefore not limited to sustainability. Its high density of chemically addressable hydroxyl groups provides a platform for tuning crosslinking, polarity, solvent affinity, and interfacial interactions. However, hydrophilicity also creates a battery-processing challenge: residual moisture, uncontrolled swelling, and batch-to-batch differences in substitution or fibril structure can strongly affect electrochemical behavior. Cellulose-derived GPEs should therefore be evaluated with the same rigor as synthetic hosts, including water content, drying history, electrolyte fraction, and long-term interfacial stability. Kim et al. developed wood-derived, charge-engineered cellulose nanofibrils (c-CNF) that combine slurry dispersion control with mechanical reinforcement in NCM811 electrodes [58]. As illustrated in Figure 7a, the binder enables NMP-free electrode fabrication through slurry casting and calendering. Quaternary ammonium groups promote electrostatic repulsion during slurry processing, suppressing particle aggregation, while hydrogen bonding and nanofibril entanglement strengthen the electrode after drying. The resulting interconnected architecture improves electrolyte infiltration and preserves transport pathways, contrasting with the aggregation, cracking, and detachment depicted for the PVDF-based counterpart (Figure 7b). This design enabled NCM811 electrodes with a mass loading of 113 mg cm^−3^ and an electrode density of 3.65 g cm^−3^, delivering an areal capacity of 22.5 mAh cm^−2^ at 0.05 C [58]. Although charge-engineered cellulose nanofibrils primarily function as electrode binders rather than GPEs, their inclusion highlights how polymer surface chemistry and mechanical network design can improve electrode integrity and electrolyte accessibility, providing complementary insights for polymer-based battery materials.

**Figure 8:**
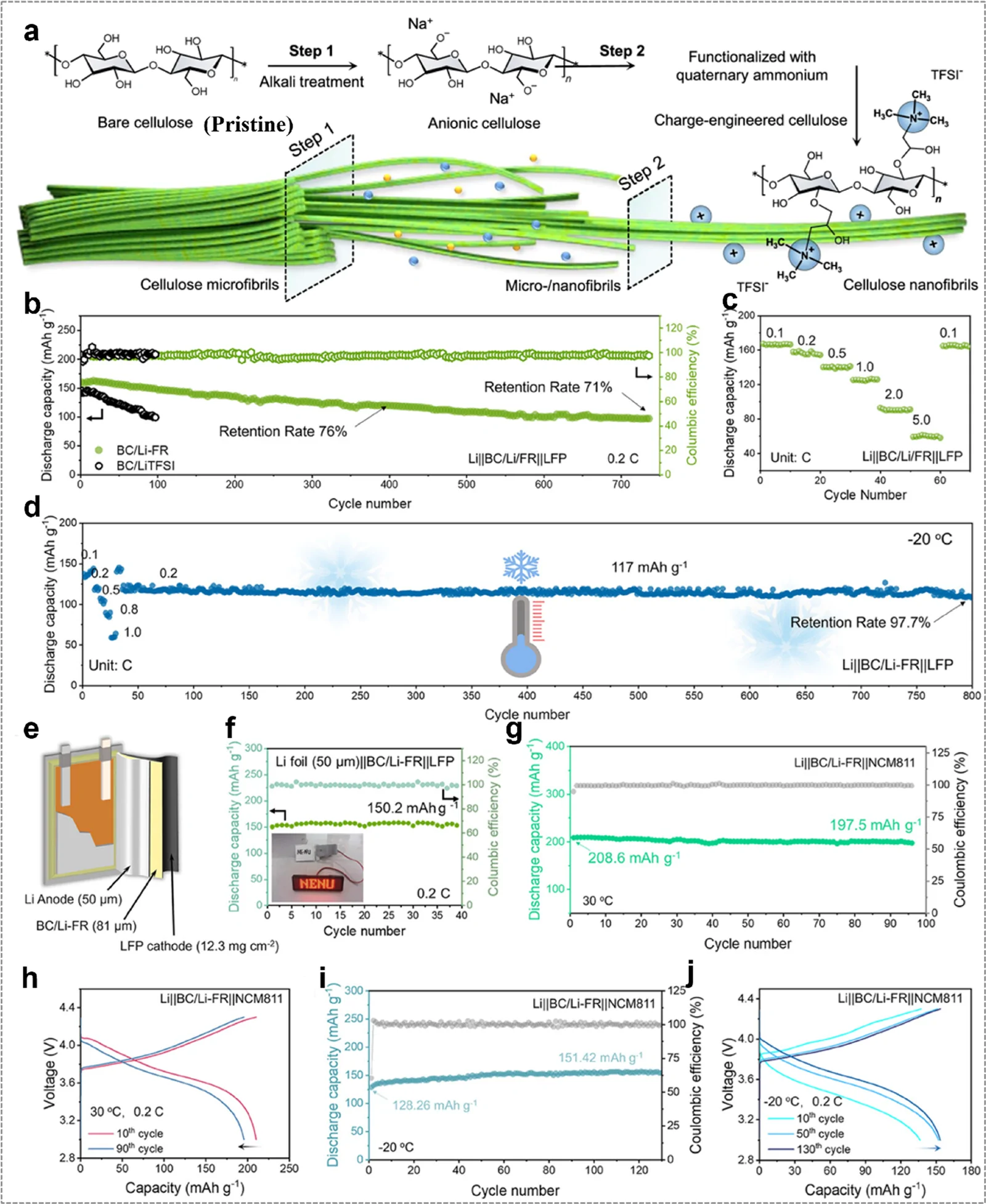
Cellulose functionalization and biomimetic electrolyte design for lithium batteries. (**a**) Preparation of charge-engineered cellulose nanofibril cathode binders. Reproduced from reference [58]. (**b**,**c**) Li||LFP cycling and rate performance with BC/Li-FR at 30 °C. (**d**) Rate and cycling behavior at −20 °C. (**e**,**f**) Li||LFP pouch-cell configuration and cycling performance. (**g**,**h**) Li||NCM811 cycling and voltage profiles at 30 °C. (**i**,**j**) Corresponding results at −20 °C. Cycling measurements in (**b**,**d**,**f**,**g**,**i**) were conducted at 0.2 C. Reproduced with permission from reference [59].

Beyond cellulose surface functionalization for electrode binding (Figure 8a) [58], biomimetic composite assembly provides a distinct strategy for developing multifunctional GPEs. Liu et al. developed a plant-cell-wall-inspired electrolyte, BC/Li-FR, combining bacterial cellulose, lignin-derived flame-retardant microspheres, and lithiated hydroxyapatite [59]. The authors attributed its performance to hydrogen-bond-mediated anion confinement, solvent-swollen transport pathways, and the formation of a LiF/Li_3_P-rich interphase. The electrolyte exhibited ionic conductivities of 4.69 mS cm^−1^ at 30 °C and 0.162 mS cm^−1^ at −20 °C, together with a reported Li^+^ transference number of 0.75. These coupled transport and interfacial properties supported prolonged low-temperature cycling. Li||LFP cells showed a reported capacity retention of 97.7% over 800 cycles at −20 °C and 0.2 C (Figure 8b–d), while Li||NCM811 cells delivered approximately 151.4 mAh g^−1^ after 130 cycles at −20 °C (Figure 8i,j). A separate Li||LFP pouch-cell demonstration maintained approximately 150.2 mAh g^−1^ over 40 cycles at 30 °C (Figure 8e–h) [59]. These results illustrate the potential of cellulose-based composite architectures for subzero operation, although the demonstrations employed lithium-metal anodes rather than conventional graphite-based LIB configurations.

### 3.5. Emerging Functional Hosts, Adaptive Networks, and Wide-Temperature Matrices

Recent GPE research increasingly designs the polymer around a specific failure mode rather than selecting a host solely for film formation. Fluorinated PEGDA-based networks have been used to combine low-temperature transport with high-voltage compatibility [60], while recent molecular-level analyses emphasize electron-withdrawing units, solvation control, Lewis acid–base interactions, hydrogen bonding, and other intermolecular interactions as direct handles for regulating oxidative stability and Li^+^ transport [61]. Fluorinated acrylate networks combined with low-volatility or plastic-crystal components have also reached millisiemens-per-centimeter conductivity while maintaining high-voltage compatibility in demanding high-Ni cathode cells [62]. The emerging design principle is thus to couple backbone chemistry, liquid-phase solvation, and interphase-forming reactions rather than treating them as independent variables. Other functional hosts emphasize flame resistance, self-healing, adhesion, or temperature-responsive behavior. Dynamic covalent or supramolecular networks can restore mechanical continuity after damage and help maintain electrode contact during repeated cycling [24,27]. Thermally triggered crosslinking and phosphorus-containing polymer chemistries can additionally reduce free-liquid mobility or radical propagation at elevated temperature [63,64,65]. Such multifunctionality is attractive when a single structural motif addresses more than one failure mode, but it also increases the burden of mechanistic verification: changes in conductivity, solvent activity, interphase composition, and thermal behavior should be separated experimentally rather than attributed generically to synergy. Emerging gel-polymer electrolytes increasingly employ functional hosts to regulate Li^+^ solvation and interfacial chemistry rather than merely immobilize liquid components. Selective polymer–solvent interactions, diversified coordination environments, and mechanically compliant networks provide complementary routes to combine efficient ion transport with electrode protection. These approaches shift electrolyte design toward the coordinated control of molecular interactions and interphase stability, particularly under temperature conditions that challenge conventional gel systems [66,67,68].

**Figure 9:**
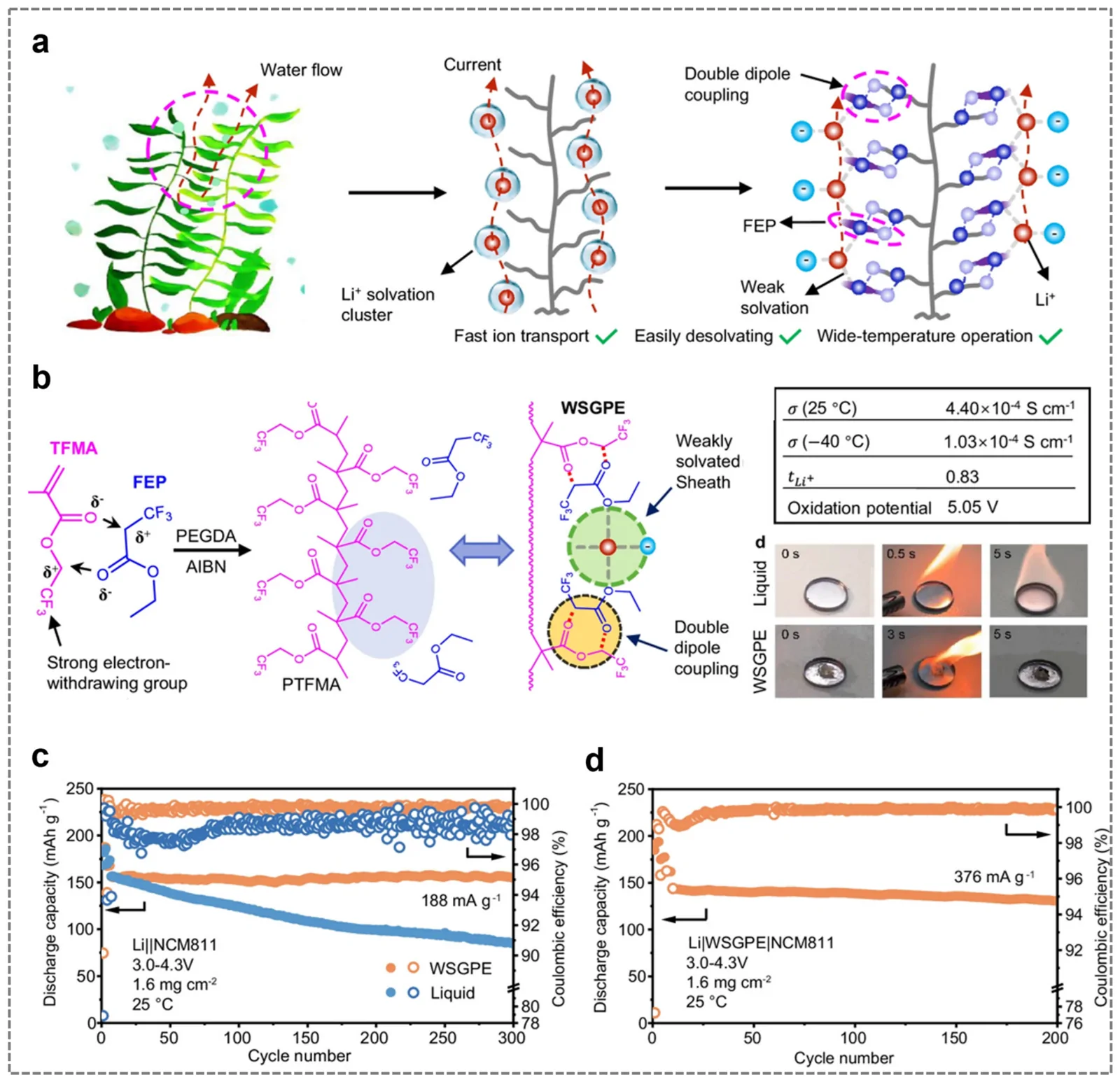
Bio-inspired polymer–solvent coupling for wide-temperature gel electrolytes. (**a**), Water-grass-inspired design illustrating double-dipole coupling and Li^+^ solvation regulation. (**b**), Molecular architecture and preparation of WSGPE, together with its electrochemical properties and comparative ignition tests. (**c**,**d**) Discharge capacity and Coulombic efficiency of Li||NCM811 cells at 188 mA g^−1^ (**c**) and 376 mA g^−1^ (**d**), measured at 25 °C within 3.0–4.3 V using a cathode loading of 1.6 mg cm^−2^. Reproduced from reference [66].

A representative example is the bioinspired weakly solvated gel-polymer electrolyte (WSGPE), comprising a brush-like poly (trifluoroethyl methacrylate) (PTFMA) framework and ethyl 3,3,3-trifluoropropanoate (FEP). Double-dipole coupling preferentially associates FEP with the polymer side chains, reducing solvent participation in the primary Li^+^ coordination shell and generating an anion-rich solvation environment (Figure 9a,b). The host therefore regulates coordination indirectly through solvent affinity, facilitating interfacial desolvation without strongly binding Li^+^ itself [66]. WSGPE exhibits ionic conductivities of 4.40 × 10^−4^ S cm^−1^ at 25 °C and 1.03 × 10^−4^ S cm^−1^ at −40 °C, with a Li^+^ transference number of 0.83. Li||NCM811 cells retain 92.5% capacity after 300 cycles at 188 mA g^−1^ and 25 °C, using a cathode loading of 1.6 mg cm^−2^ (Figure 9c,d). Operation from −30 to 80 °C further demonstrates the benefits of combining solvation regulation with stable electrode–electrolyte interphases [66].

**Figure 10:**
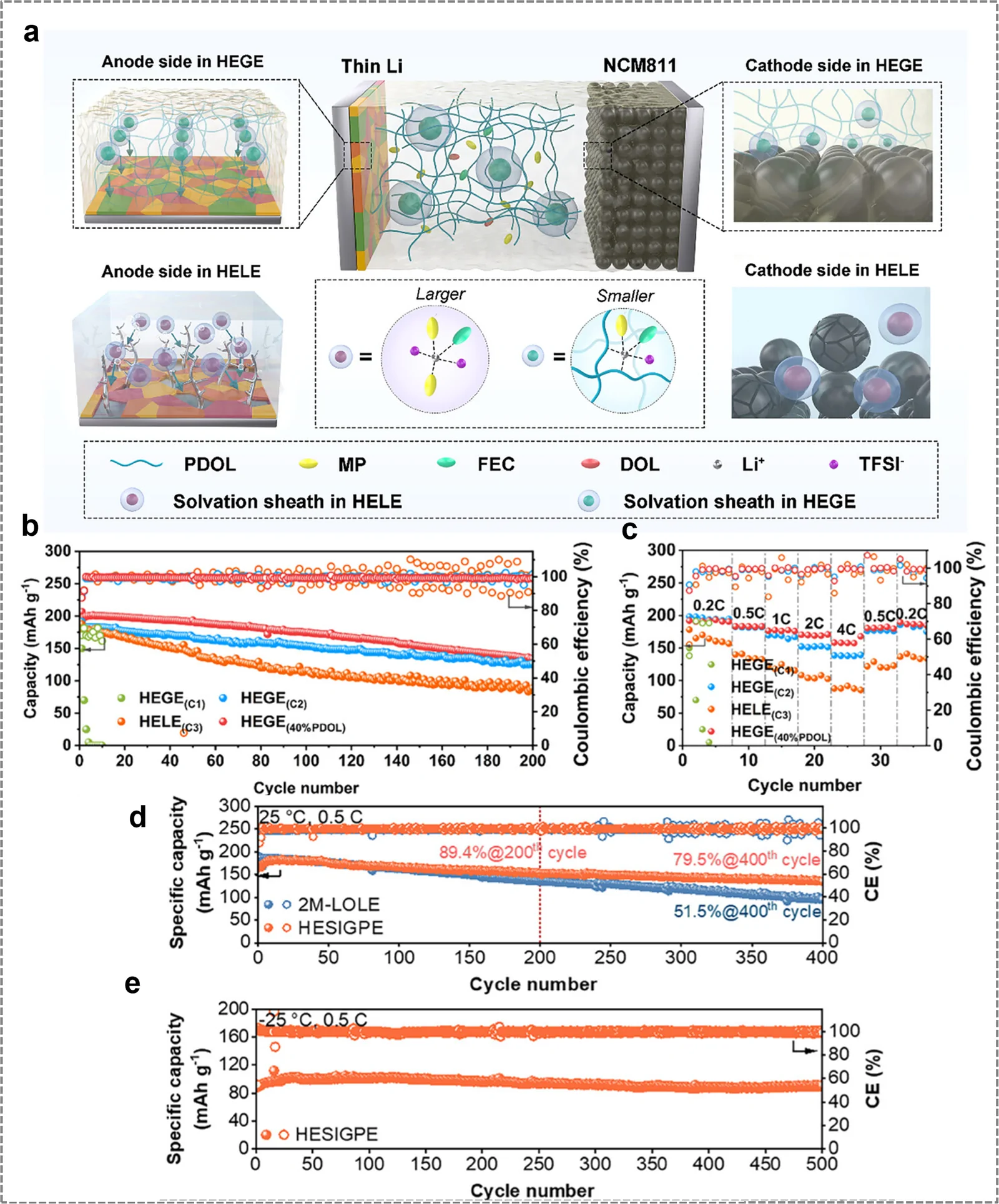
High-entropy gel hosts for solvation regulation and interfacial protection. (**a**) Schematic comparison of Li^+^ solvation and electrode interfaces in PDOL-containing HEGE and its liquid counterpart, HELE. (**b**) Li||NCM811 cycling performance at 25 °C and 0.5 C. (**c**) Rate capability with HEGE and the indicated controls. Reproduced with permission from reference [67]. (**d**) Cycling comparison of HESIGPE and the 2M-LOLE liquid reference at 25 °C and 0.5 C. (**e**) Li||NCM811 cycling with HESIGPE at −25 °C and 0.5 C over 500 cycles. Reproduced with permission from reference [68].

High-entropy gel electrolytes extend this concept through coordination diversification. In HEGE, LiDFOB-initiated polymerization of dioxolane produces a poly(dioxolane) (PDOL) matrix within a multicomponent electrolyte containing methyl propionate and fluoroethylene carbonate (Figure 10a). Unlike the predominantly solvent-binding PTFMA host, PDOL directly participates in Li^+^ coordination, decreasing solvent involvement in the solvation shell. Molecular-dynamics simulations and diffusion-ordered NMR associate the optimized component diversity and binding interactions with smaller Li^+^ clusters and enhanced transport [67]. The optimized HEGE(40%PDOL) achieves ionic conductivities of 9.40 mS cm^−1^ at 25 °C and 4.42 mS cm^−1^ at −25 °C. Its Li||NCM811 cells deliver 157.8 mAh g^−1^ at 4 C (Figure 10b,c) and retain 75.4% capacity after 500 cycles at −25 °C and 0.5 C, illustrating the combined importance of coordination chemistry and low-temperature transport [67].

HESIGPE further integrates chemical protection into a high-entropy single-ion-conducting polymer host. Its flexible polydimethylsiloxane-based architecture incorporates tethered anionic groups and ether-containing side chains, combining ion-transport functionality with an elastic network. Competitive coordination reduces solvent participation around Li^+^, while siloxane and amine functionalities provide HF-scavenging capability, supported by ^19^F NMR measurements [68]. HESIGPE maintains an ionic conductivity of 1.62 mS cm^−1^ at −25 °C and enables 79.5% capacity retention after 400 cycles at 25 °C and 0.5 C, compared with 51.5% for the liquid reference (Figure 10d). Extended cycling at −25 °C further demonstrates its low-temperature applicability (Figure 10e). Importantly, the study attributes the improved long-term response primarily to interfacial stabilization and suppressed side reactions [68].

## 4. Strategies for Improving GPE Performance

### 4.1. Polymer Blending and Network Crosslinking

Polymer blending is a direct way to combine complementary functions within one membrane. A mechanically robust but crystalline framework can be blended with a softer, more amorphous, or more electrolyte-affine polymer to increase uptake and reduce transport barriers. Representative combinations include PVDF-HFP/PEO, PVDF/cellulose derivatives, PAN/PVA, and cellulose/PEG systems [18,26,32,40,50]. The benefit is greatest when the components form a stable, continuous morphology. Uncontrolled phase separation can instead create isolated liquid pockets, nonuniform current pathways, or mechanically weak regions. Chemical crosslinking is particularly valuable for high-liquid-content GPEs because it suppresses flow, leakage, and macroscopic swelling. The optimum network, however, is rarely the most highly crosslinked one. Increasing crosslink density reduces chain mobility, pore swelling, and sometimes salt dissociation, thereby increasing ionic resistance. High-performance systems increasingly use flexible spacers, semi-interpenetrating networks, or dual-network architectures in which a mechanically load-bearing skeleton is partially decoupled from liquid-rich ion-conduction domains [25,69,70,71,72]. This decoupling concept is more general than any specific polymer chemistry and should be considered a central design rule for next-generation GPEs.

### 4.2. Inorganic and Nanostructured Fillers

Inorganic fillers can modify GPE behavior through mechanical reinforcement, crystallinity suppression, Lewis acid–base interactions, interfacial ion transport, and solvent retention. Oxides such as SiO_2_ can increase rigidity and disrupt polymer ordering, whereas ion-conducting ceramics such as LAGP may introduce additional pathways or alter local electric-field and coordination environments [25,73,74,75]. The effect is highly dependent on particle size, surface chemistry, loading, and dispersion. Agglomerated particles can increase tortuosity, generate brittle defects, and reduce effective conducting volume, so a higher filler fraction does not necessarily translate into better electrochemical performance. High-surface area or chemically functionalized fillers are particularly useful when their interfaces are deliberately coupled to the polymer network. Methacrylate-functionalized mesoporous SiO_2_, for example, can serve simultaneously as a reinforcing phase and a reactive crosslinking center [25]. Other multifunctional fillers combine mechanical support with thermal conduction, species adsorption, or interphase regulation [73]. Future filler studies should therefore quantify dispersion and interfacial chemistry rather than treating the inorganic phase as an inert additive; otherwise, improvements in conductivity or cycling can be difficult to distinguish from changes in porosity or liquid uptake.

### 4.3. Ionic Liquids, Flame-Retardant Components, and Functional Additives

Ionic liquids, plastic crystals, and low-volatility cosolvents can reduce vapor pressure and extend the operating-temperature range while preserving a mobile ion-conduction phase [30,54]. Their introduction, however, changes viscosity, solvation structure, salt activity, and electrode interphase chemistry. Consequently, a low-flammability liquid phase should not be evaluated only by bulk conductivity or ignition behavior; its reduction and oxidation products must also be compatible with graphite, Si-containing anodes, and high-voltage cathodes. Functional additives provide a second route to couple safety and interfacial stabilization. Hindered-amine/cyclophosphazene additives have been used to suppress radical propagation and improve high-voltage compatibility, while boron- or fluorine-containing additives can promote protective cathode-electrolyte interphases [76,77]. More recent LIB-focused work extends this strategy to phosphazene-containing in situ gels and EC-less low-reactivity formulations that directly reduce heat release and electrode-to-electrode crosstalk during thermal abuse [78,79]. These results reinforce a key point: the polymer skeleton, retained solvent, salt, and additive package must be treated as one electrolyte system because each component can alter flammability, solvation, interphase composition, and transport. Flame-retardant components can contribute beyond combustion suppression by simultaneously regulating Li^+^ coordination and electrode–electrolyte interphases. Two complementary approaches illustrate this multifunctionality: incorporating weakly coordinating phosphazene molecules into polyether gels and covalently integrating phosphorus-containing flame retardants into crosslinked networks. These strategies connect electrolyte safety with molecular interactions and polymer architecture, rather than treating flame resistance as an isolated formulation objective [80,81]. In the PDFP electrolyte, in situ polymerization of 1,3-dioxolane produces a polyether framework containing fluoroethylene carbonate (FEC) and ethoxy(pentafluoro)cyclotriphosphazene (NPCF). NPCF combines flame-retardant functionality with weak coordination toward Li^+^, thereby modifying solvation without becoming a dominant first-shell ligand [80]. Molecular-dynamics simulations indicate that NPCF remains outside the primary solvation shell while reducing the FEC coordination number from 1.21 to 1.01 and increasing PF_6_^−^ participation. This redistribution favors anion-involved interfacial chemistry, while NPCF decomposition contributes phosphorus- and nitrogen-containing species to the cathode electrolyte interphase. Thus, its role extends from suppressing combustion to directing the chemistry of electrode passivation [80]. The optimized PDFP-1 formulation exhibits an ionic conductivity of 1.15 × 10^−4^ S cm^−1^ at 25 °C and a Li^+^ transference number of 0.633. Importantly, increasing the NPCF content does not continuously improve performance: excessive addition produces salt precipitation and lower conductivity, highlighting the need to balance flame-retardant content with electrolyte homogeneity and ion transport [80]. This compositional dependence is reflected in the cycling and rate responses of Li||NCM85 cells. PDFP-1 provides better cycling persistence than PDFP-2 and PDFP-3, with a reported capacity retention of 60.1% after 200 cycles at 1 C within 3.0–4.5 V. At 50 °C, the cell retains 56.8% after 95 cycles at 0.3 C within 2.7–4.3 V. These results demonstrate improved compatibility under demanding conditions, although the remaining capacity decay indicates that interfacial degradation is mitigated rather than eliminated [80]. A different strategy immobilizes the flame-retardant functionality within the polymer network. TD-GPE is prepared by copolymerizing tri(acryloyloxyethyl) phosphate (TAEP), diethyl vinylphosphonate (DEVP), and pentaerythritol tetraacrylate in a carbonate electrolyte. The authors attribute its effectiveness to complementary gas- and condensed-phase mechanisms: DEVP-derived phosphorus radicals interrupt combustion chain reactions, whereas TAEP promotes char formation that restricts heat and oxygen transfer. The resulting gel exhibits a reported burning duration of only 0.9 s in the direct-flame test [81]. This flame resistance is accompanied by an ionic conductivity of 0.51 mS cm^−1^ at 25 °C, compared with 0.64 mS cm^−1^ for the liquid reference, showing that incorporation of reactive flame retardants can preserve substantial ionic transport [81]. Crucially, electrolyte self-extinguishing behavior must be distinguished from suppression of cell-level thermal hazards. The liquid-electrolyte NCM811||graphite pouch cell undergoes swelling, sustained combustion, and jet fire during the illustrated thermal-abuse sequence. In the corresponding TD-GPE experiment reported in the full study, jet fire and electrolyte leakage were suppressed, although smoke and transient sparks remained [81]. Moreover, TD-GPE delayed but did not prevent thermal runaway, which occurred after a second heating stage [81]. Together, these studies emphasize that multifunctional additives should be assessed through both electrochemical durability and device-level abuse testing: favorable solvation chemistry and short burning durations are valuable, but neither alone establishes comprehensive battery safety.

### 4.4. Processing Routes: Solution Casting, Electrospinning, and In Situ Polymerization

Solution casting remains the simplest route for preparing free-standing GPE membranes. Polymer, salt, filler, and plasticizer or precursor are dissolved or dispersed, cast into a film, and dried before electrolyte activation, when required. The approach is conceptually scalable and provides direct control over membrane thickness, but residual solvent, drying energy, pore morphology, and subsequent electrolyte uptake can introduce substantial variability [16,82,83]. For practical manufacturing, solvent choice and recovery, membrane uniformity over large areas, and compatibility with existing separator-handling equipment are as important as laboratory-scale conductivity. Electrospinning creates interconnected nanofibrous scaffolds with high porosity and surface area. These structures can absorb large amounts of liquid electrolytes while retaining a mechanically continuous separator framework, and have been demonstrated using PVDF-HFP, PAN, and multilayer architectures [40,84,85,86]. Their principal advantages are short wetting distances and tunable pore structure; their practical challenges are fiber and pore-size uniformity, thickness control, solvent handling, and high-throughput production. In situ polymerization is currently one of the most important processing routes for translating GPEs toward practical LIBs. A low-viscosity precursor can infiltrate electrode pores before thermal, UV, or chemical polymerization, allowing the gel network to form directly at the cathode and anode surfaces and thereby reduce dry contact regions and interfacial impedance [60,70,71]. Recent studies demonstrate that this approach can support high-current-density graphite||NCM811 full cells [40], Ah-level NCM811/graphite pouch cells with LiF-rich interfaces [87], and graphite Si/high-Ni quasi-solid pouch cells with improved thermal-abuse tolerance [88]. The same processing advantage creates new control requirements: monomer conversion, initiator residues, polymerization shrinkage, gel fraction, precursor viscosity, wetting time, and compatibility with formation protocols must be reproducible. In situ polymerization should therefore be evaluated as a manufacturing process, not only as a materials-synthesis method.

### 4.5. Self-Healing and Multifunctional GPEs

Table 2 lists the GPE systems and reported cell-level performance. Multifunctional GPEs extend the electrolyte beyond the conventional roles of ion conductor and separator. Dynamic hydrogen bonds, reversible covalent networks, and adaptive polymer architectures can recover mechanical continuity after damage and maintain interfacial contact during cycling [24,27]. This concept is especially relevant to Si-containing anodes, where repeated volume changes continuously perturb the electrolyte/electrode interface. Multiple-hydrogen-bond networks have recently enabled pressure-free cycling of Ah-scale SiO_x_/graphite||NCM811 pouch cells, illustrating how self-healing chemistry can become a cell-level mechanical function rather than only a cut-and-heal demonstration [57]. The most useful multifunctionality occurs when one chemical motif addresses coupled failure modes. Fluorinated groups can modify oxidative stability while contributing to LiF-rich interphases; phosphorus-containing groups can improve flame resistance and alter interphase chemistry; fillers can reinforce the network while adsorbing reactive species; and thermally activated crosslinking can reduce ion mobility during abuse [25,63,64,65,70,78,79]. The principal risk is formulation complexity. Every added component introduces cost, possible side reactions, and a new source of process variation, so multifunctional designs should be justified by quantitatively demonstrated benefits at the full-cell level.

## 5. Applications for High-Performance Lithium-Ion Batteries

### 5.1. LiFePO_4_-Based Cells

LiFePO_4_ (LFP) is frequently used as an initial platform for evaluating GPEs because its moderate operating voltage and robust olivine structure reduce the severity of electrolyte oxidation at the cathode. PVDF, PEO, cellulose-, and composite GPEs can therefore be compared with less interference from high-voltage cathode degradation [29,30,31,32,33,34,40,50,74]. In optimized formulations, LFP cells often approach the capacity of liquid-electrolyte controls while providing improved dimensional stability, solvent immobilization, or reduced flammability. These experiments are useful for screening transport and mechanical properties, but they do not by themselves establish compatibility with high-energy LIB chemistries. For LIB relevance, the critical step is to move from Li|LFP model cells to graphite-containing full cells and realistic safety tests. A three-dimensional interpenetrating nonflammable GPE reported in 2024 was evaluated not only in Li/LFP and Li/NCM523 configurations but also in LFP/graphite cells, and pouch-cell testing showed a substantial reduction in maximum surface temperature during the reported safety test [91]. This distinction is important because graphite introduces reduction chemistry, SEI formation, initial Coulombic-efficiency loss, and pore-wetting constraints that are absent when Li metal is used as the counter electrode. LFP systems remain particularly useful for studying mechanical durability, sustainable polymer hosts, and separator-level safety because the cathode itself is comparatively stable. However, future LFP/GPE studies aimed at practical LIBs should increasingly report full-cell balancing, realistic areal loading, electrolyte quantity, formation conditions, and thermal-abuse behavior. Without these parameters, long cycle life in a low-loading Li-metal coin cell can overstate the practical maturity of the electrolyte.

### 5.2. Ni-Rich High-Voltage Cathodes

Ni-rich layered cathodes create a substantially more challenging electrolyte environment than LiFePO_4_ (LFP) because increasing Ni content enhances practical capacity while also promoting surface reactivity at high states of charge, transition-metal dissolution, lattice oxygen activity, gas generation, and electrolyte oxidation. Consequently, GPEs must simultaneously provide efficient Li^+^ transport and form a stable cathode–electrolyte interphase (CEI) that limits continuous solvent and polymer decomposition [45,61,92]. Although a wide electrochemical stability window measured by linear sweep voltammetry (LSV) is often considered an indicator of high-voltage compatibility, it does not necessarily reflect long-term interfacial stability under practical operating conditions. Therefore, sustained cycling performance, impedance evolution, gas generation, and post-cycling surface characterization are essential for evaluating electrolyte compatibility with Ni-rich cathodes. Various GPE design strategies have been investigated for NCM622, NCM811, NCA, and other Ni-rich layered cathodes, including fluorinated polymer networks, nitrate-containing or dual-reactive crosslinked systems, chelating polymers, flame-retardant additives, and multilayer electrolyte architectures [60,64,65,70]. These approaches demonstrate that electrochemical performance is governed not only by the properties of the bulk polymer matrix but also by the interfacial chemistry developed during battery operation. In particular, electron-withdrawing fluorinated groups, regulated Li^+^ solvation structures, anion-derived interphases, and radical-scavenging or phosphorus-containing functional groups can mitigate electrolyte oxidation and gas evolution, thereby improving interfacial stability [61,62,63,75,76,77]. Nevertheless, many studies evaluating GPE compatibility with high-voltage cathodes employ lithium-metal anodes. Although these configurations provide valuable insights into cathode-side degradation and electrolyte stability, their electrochemical performance cannot be directly interpreted as evidence of practical lithium-ion battery performance.

Recent investigations using graphite-based full cells have provided stronger evidence of the practical applicability of GPEs. For example, an in situ GPE incorporating CF_3_-rich ion-transport domains and secondary-amine-based anion-binding sites exhibited an ionic conductivity of 2.6 mS cm^−1^ and a Li^+^ transference number of 0.67. The corresponding graphite||NCM811 full cells retained 73.2% of their initial capacity after 300 cycles at 3 C (1.8 mA cm^−2^) [86]. Similarly, a PEGDMA-based GPE containing ethyl difluoroacetate was designed to promote the formation of LiF-rich electrode interphases and was successfully implemented in Ah-scale NCM811/graphite pouch cells. These cells achieved an energy density of 250 Wh kg^−1^ and maintained 94.0% capacity retention after 400 cycles at 45 °C under the reported testing conditions [87]. These findings highlight the importance of integrating ion-transport regulation with interfacial stabilization and validating the resulting improvements in practical graphite-based full-cell configurations.

In addition to electrochemical performance, thermal safety remains a critical consideration for Ni-rich cathodes because highly delithiated layered oxides can undergo exothermic reactions with organic electrolyte components under abusive conditions. GPEs incorporating radical-scavenging flame retardants, phosphorus-containing polymer networks, and fluorinated components have demonstrated improved ignition resistance and high-voltage cycling stability [62,63,64,65,78]. More recently, safety evaluations have extended beyond conventional flame-resistance tests to include direct assessments of thermal runaway in pouch cells. For instance, low-reactivity, ethylene carbonate (EC)-free GPE formulations have been shown to reduce electrode–electrolyte heat release and lower the maximum temperature during thermal runaway while maintaining electrochemical performance [79]. Such cell-level evaluations provide a more comprehensive assessment of electrolyte safety than measurements of isolated polymer thermal stability. Collectively, these studies emphasize that the development of GPEs for Ni-rich cathodes requires coordinated optimization of ionic transport, interfacial stability, electrochemical durability, and thermal safety, supported by validation under practical full-cell operating conditions.

Recent full-cell studies provide stronger evidence of practical relevance. An in situ GPE containing CF_3_-rich transport domains and secondary-amine anion-binding sites delivered 2.6 mS cm^−1^ conductivity and a Li^+^ transference number of 0.67; graphite||NCM811 full cells retained 73.2% of capacity after 300 cycles at 3 C (1.8 mA cm^−2^) [86]. A PEGDMA-based GPE using ethyl difluoroacetate to generate LiF-rich interfaces was implemented in Ah-level NCM811/graphite pouch cells, reaching 250 Wh kg^−1^ and 94.0% capacity retention after 400 cycles at 45 °C in the reported configuration [87]. These examples illustrate the direction needed for high-Ni GPE research: transport and interphase design must be demonstrated together in graphite-based full cells. Safety-oriented designs are especially relevant to Ni-rich cathodes because highly delithiated layered oxides and combustible organic phases can react exothermically during abuse. Radical-scavenging flame-retardant GPEs, phosphorus-containing networks, and nonflammable fluorinated gels have shown improved ignition resistance and high-voltage cycling [62,63,64,65,78]. More recent pouch-cell work goes beyond flame tests by measuring thermal-runaway behavior directly, showing that low-reactivity EC-less GPEs can reduce electrode/electrolyte heat release and lower the peak temperature during thermal runaway while maintaining electrochemical performance [79]. Such measurements are more informative for practical safety than thermal stability of the isolated polymer alone.

Stabilizing Ni-rich cathodes requires gel-polymer electrolytes to address not only electrolyte decomposition but also transition-metal dissolution and cathode–anode cross-talk. These processes become particularly consequential when Ni-rich cathodes are paired with silicon-containing anodes, where interfacial instability accelerates the loss of cyclable lithium. Chemically protective polymer networks and spatially differentiated electrolyte architectures offer complementary approaches to stabilizing both electrode interfaces [92,93,94]. Bai et al. combined an in situ-polymerized poly (vinylene carbonate) matrix (PVCM) containing lithium difluoro(oxalato)borate (LiDFOB) with an engineered Si/C@C graphite anode and NMC811 cathode. The carbon-confined silicon architecture accommodates mechanical deformation, while the infiltrating gel provides interfacial protection. PVCM carbonyl groups and LiDFOB-derived species coordinate transition-metal ions, complementing the formation of a cathode electrolyte interphase (CEI) containing LiF, B-O, and B-F species [90]. This suppression of crosstalk is supported by elemental analysis: after 500 cycles, nickel and manganese accumulation at the anode decreased from 226 and 118 ppm in the liquid-electrolyte system to 43 and 27 ppm with the gel, respectively. At the device level, the nominal 2.7 Ah pouch cell achieved a reported capacity retention of 88.7% after 2000 cycles at 0.5 C, compared with 79.4% for the corresponding liquid-electrolyte cell, together with a specific energy of 325.9 Wh kg^−1^ based on the whole pouch-cell mass. These measurements used a full-cell voltage window of 3.0–4.2 V and an N/P ratio of approximately 1.07 [90]. Operando X-ray diffraction additionally revealed improved recovery of the NMC811 lattice parameters in the integrated GPE configuration. The results therefore connect long-term durability to coordinated electrode engineering and interfacial stabilization, rather than to electrolyte modification alone [90]. A complementary strategy spatially separates cathode- and anode-facing functionalities. Zhu et al. developed an asymmetric, electrospun PVDF-HFP gel containing barium titanate (BTO) on the cathode-facing side and hexagonal boron nitride nanosheets (h-BNNS) on the lithium-facing side. The authors associate BTO polarization with regulation of interfacial Li^+^ distribution and CEI stabilization, whereas Lewis-acidic boron sites in h-BNNS interact with PF_6_^−^ to reduce anion mobility and promote more uniform lithium deposition. Importantly, the bilayer arrangement also avoids direct contact between BTO and reactive lithium metal [93,94]. The optimized c-GPE-2 exhibits an ionic conductivity of 3.77 mS cm^−1^, while microscopy and spectroscopy support a thinner, more uniform CEI and reduced carbonate decomposition. Its oxidation onset remains approximately 4.5 V, like that of the unfilled gel, emphasizing interfacial regulation rather than substantial expansion of the measured stability window [94]. Although the principal electrochemical panels concern NCM523, the architecture was also evaluated with Ni-rich NCM811. The NCM523 cells deliver 99 mAh g^−1^ at 10 C and retain 80% capacity after 100 cycles at 2 C; the NCM811 cells provide an initial capacity of 186.4 mAh g^−1^ and 81% retention after 100 cycles at 0.5 C [94].

### 5.3. Graphite and Si-Containing Full Cells: Practical LIB Validation

Graphite and Si-containing full cells are essential for a review centered on LIBs because they reveal whether a GPE is compatible with practical negative electrodes. Graphite requires a stable, electronically insulating SEI and high initial Coulombic efficiency, while Si-containing anodes additionally undergo large volume changes that repeatedly stress the interphase and can expel or redistribute electrolyte. A GPE that performs well in Li||NCM or Li||LFP cells may therefore fail in a true full cell because of reduction chemistry, wetting limitations, electrolyte inventory, or mechanical loss of contact. Recent studies provide several important demonstrations. In situ polymerized GPEs have enabled graphite||NCM811 cells to cycle at high current density [86]. A LiF-forming PEGDMA/DFEA GPE was implemented in Ah-level NCM811/graphite pouch cells with 250 Wh kg^−1^ energy density and 94.0% retention after 400 cycles at 45 °C [87]. Separately, an EC-free liquid electrolyte combined with in situ polymerization produced Ah-level LiNi_0.83_Co_0.11_Mn_0.06_O_2_||graphite Si quasi-solid cells with substantially improved hot-box and thermal-runaway tolerance relative to liquid-electrolyte controls [88]. These studies are particularly valuable because they test electrolyte performance under coupled cathode, anode, thermal, and cell-format constraints. Si-containing cells place an additional premium on adaptive interface mechanics. The 2024 in situ polymerization strategy reported for Si-containing anode/Ni-rich cathode pouch cells achieved 325.9 Wh kg^−1^ and 88.7% retention over 2000 cycles in the reported 2.7 Ah configuration [90]. More recently, a multiple-hydrogen-bond GPE based on in situ copolymerization was designed to limit SiO_x_ expansion and preserve electrolyte contact; Ah-scale SiO_x_/graphite||NCM811 pouch cells were operated for 1000 cycles without external stack pressure [57]. Together, these results suggest that successful Si-compatible GPEs must combine interphase chemistry with mechanically adaptive adhesion. Simply increasing modulus is unlikely to be sufficient because the electrolyte must accommodate repeated local strain while preserving ion-conduction pathways.

### 5.4. Wide-Temperature Operation, Thermal Safety, and Abuse Tolerance

The term gel does not by itself imply a safe electrolyte. If a GPE contains a large fraction of conventional carbonate solvent, gelation may reduce leakage while leaving substantial chemical fuel available during thermal runaway. Meaningful safety assessment therefore requires direct measurements such as self-extinguishing time, thermal shrinkage, calorimetry, gas generation, nail or penetration tests, overcharge, hot-box testing, or accelerating-rate calorimetry, preferably at the complete-cell level [79,88,91]. Recent practical studies show that in situ polymer networks can improve safety by more than immobilizing liquid: they can suppress electrode and electrolyte exothermic reactions, reduce cathode–anode crosstalk, and alter the temperature at which runaway reactions accelerate [79,88]. Wide-temperature operation is another coupled problem. At low temperature, solvent viscosity rises, salt dissociation and desolvation kinetics change, and interfacial charge transfer can dominate polarization. At high temperature, polymer softening, solvent migration, gas-forming side reactions, and interphase dissolution accelerate. Recent reviews emphasize that wide-temperature GPE design must jointly optimize the mobile phase, polymer dynamics, solvation structure, and interphase chemistry rather than relying on a single low-glass-transition-temperature polymer [95]. Fluorinated PEGDA systems, succinonitrile-containing gels, and ionic-liquid or low-volatility formulations illustrate possible approaches [54,60,62,96,97]. For practical claims, conductivity should be reported across the stated temperature range and accompanied by full-cell cycling or abuse data at the same temperatures.

## 6. Current Challenges and Future Perspectives

### 6.1. Conductivity-Mechanical Strength Trade-Off

The most persistent GPE design challenge is the coupling between liquid-like transport and solid-like mechanical integrity. Higher solvent content, larger free volume, lower crystallinity, and increased chain mobility generally improve ionic conductivity but reduce modulus, dimensional stability, and resistance to solvent migration. Higher crosslink density or filler loading can reverse the mechanical trend but may increase tortuosity and restrict segmental or solvent motion [16,26,44]. The most promising strategy is therefore architectural rather than purely compositional: dual networks, reinforced porous scaffolds, phase-separated ion-conducting domains, and moderate crosslink densities can partially decouple load bearing from ion transport [25,26,27,56,72]. Future studies should report conductivity and mechanical properties on the same fully activated formulation, because dry-membrane strength and gel-state strength are not interchangeable.

### 6.2. Electrode–Electrolyte Interface Stability

Bulk conductivity cannot predict cell lifetime when interfacial reactions dominate. High-Ni cathodes require oxidative stability and a thin, ion-conductive CEI, whereas graphite and Si-containing anodes require reduction products that suppress continuous solvent consumption and gas generation. In situ polymerization is attractive because it improves physical contact before cycling begins, but conformal contact does not guarantee chemical stability [45,71,77,98,99,100]. Current research increasingly combines network design with fluorinated groups, controlled solvation, chelating sites, anion-binding motifs, or additives that deliberately direct SEI/CEI formation [86,87]. The next step is to correlate these chemistries with time-resolved impedance, gas evolution, depth-resolved surface analysis, and cross-sectional morphology rather than inferring interface stabilization from capacity retention alone.

### 6.3. Long-Term Electrolyte Retention, Temperature Tolerance, and Safety

Because GPE contains a liquid or plasticizing phase, long-term durability depends on maintaining its composition as well as its polymer structure. Evaporation, solvent migration, swelling, phase separation, and redistribution between electrode and separator regions can progressively change conductivity, salt concentration, and mechanical response. These processes accelerate during thermal cycling and high-temperature storage. Stronger polymer–solvent interactions, low-volatility plasticizers, ionic liquids, and chemically crosslinked networks can reduce these losses, but may increase viscosity or interfacial resistance [43,51,95]. Long-term studies should therefore quantify mass or solvent retention and impedance evolution, not only discharge capacity. Safety must likewise be evaluated for the complete activated gel and preferably the complete cell. Thermogravimetric stability of the dry polymer is insufficient if the final GPE still contains a flammable liquid phase. Recent NCM/graphite pouch-cell studies demonstrate more rigorous approaches, including hot-box tests, overcharge or penetration, heat-release analysis, and direct measurement of thermal-runaway peak temperature [87,88]. These methods reveal whether gelation changes the reaction pathway and thermal feedback of the cell, rather than merely delaying leakage or ignition in an isolated electrolyte sample.

### 6.4. Processing, Scalability, and Reproducibility

Manufacturing compatibility remains less mature than laboratory electrochemical performance. Solution casting can require large quantities of volatile solvent and long drying times; electrospinning adds equipment and throughput constraints; and in situ polymerization introduces monomer, initiator, curing, and quality-control steps inside the cell [16,41,101]. The practical attractiveness of in situ gelation is that it can use conventional electrolyte-filling hardware and improve pore infiltration, but this advantage will only be realized if precursor viscosity, pot life, conversion, gel fraction, shrinkage, residual monomer, and curing temperature are tightly controlled. Recent Ah-scale demonstrations show that in situ GPE processing can be compatible with pouch cells, but reproducibility and long-term storage stability remain critical translation questions [57,78,79,87,102,103]. Comparison across published GPEs is further complicated by inconsistent reporting of membrane thickness, liquid fraction, salt concentration, electrode loading, N/P ratio, stack pressure, formation protocol, voltage window, temperature, and electrolyte quantity. A high conductivity measured in a thin, solvent-rich membrane cannot be directly compared with a mechanically robust low-liquid gel tested under different conditions. Studies claiming relevance to high-performance LIBs should, at minimum, report conductivity as a function of temperature, Li^+^ transference when mechanistically important, gel or liquid fraction, mechanical properties in the activated state, electrochemical-stability methodology, interfacial impedance, electrode loading, and complete full-cell conditions. Pouch-cell validation is particularly valuable because it exposes gas generation, electrolyte redistribution, thermal behavior, and mechanical constraints that coin cells can obscure.

### 6.5. Research Priorities Toward Practical LIBs

The next generation of GPEs should be designed through integrated molecular, interfacial, and cell engineering. Major challenges in GPE development are presented in Table 3. Polymer backbones and side groups should provide the required oxidation and reduction stability; network topology should balance liquid retention with compliance; the liquid phase and salt should be selected for favorable solvation and temperature response; and additives or fillers should target identified parasitic reactions rather than being added empirically [61,72,95,104,105,106,107]. In situ polymerization is likely to remain important because it addresses interfacial conformity and manufacturing compatibility simultaneously, but future work should establish robust curing windows and quantify residual species. Data-driven or high-throughput approaches may also help navigate the large formulation space, provided that experimentally meaningful descriptors such as liquid fraction, coordination environment, modulus, and interfacial resistance are included. Most importantly, practical validation should become part of the materials-design loop rather than a final demonstration. Recent graphite||NCM811, NCM811||graphite pouch, and SiO_x_/graphite||NCM811 pouch-cell studies show that promising GPE concepts can survive full-cell constraints when transport, interphase chemistry, and mechanics are engineered together [57,86,87,88]. Future studies should increasingly test realistic electrode loadings, limited electrolyte inventory, controlled N/P ratio, elevated and subzero temperatures, storage, fast-charge conditions, and abuse scenarios. Sustainable polymer hosts and low-toxicity processing remain valuable goals, but their significance will depend on whether they can meet the same electrochemical purity, moisture control, and manufacturing consistency required of conventional battery materials.

## 7. Conclusions

Gel polymer electrolytes provide a versatile route to combine liquid-like ion transport and wetting with the dimensional stability and functional tunability of polymer networks. Their performance is governed by the coupled behavior of the polymer framework, liquid phase, lithium salt, additives, and electrode interfaces; consequently, no single polymer host is universally superior. PVDF/PVDF-HFP systems provide chemical robustness and strong film formation, PEO offers effective Li^+^ coordination but requires control of crystallinity and oxidative stability, PAN and methacrylate networks provide tunable polarity and crosslinking, and cellulose-derived hosts introduce mechanically strong and potentially sustainable alternatives. Recent progress shows that the field is moving beyond membrane-level optimization. Blending, crosslinking, functional fillers, flame-retardant chemistry, self-healing networks, and especially in situ polymerization are increasingly used to control electrode contact and interphase formation in addition to bulk transport. The most significant evidence comes from recent high-Ni/graphite and high-Ni/Si graphite full cells and Ah-scale pouch cells, where GPEs have enabled high-rate cycling, improved high-temperature durability, pressure-free Si operation, and measurable improvements in thermal-abuse response. These studies also demonstrate why Li-metal half-cell data, although mechanistically useful, should not be used alone to claim practical LIB performance. Future progress will depend on balancing conductivity, Li^+^ selectivity, mechanical compliance, solvent retention, interphase chemistry, safety, and manufacturability within the same formulation. Standardized reporting and realistic full-cell validation will be essential for distinguishing genuine electrolyte advances from favorable test conditions. The most promising GPEs are therefore likely to emerge from integrated electrolyte–electrode process design, in which the network is engineered not only to immobilize liquid but also to regulate solvation, preserve contact, direct SEI/CEI chemistry, and remain compatible with scalable cell fabrication.

## Figures and Tables

**Table 1:** Representative polymer matrices used in gel polymer electrolytes.

Polymer Matrix	Structural Feature	Main Advantages	Typical Limitations
PVDF/PVDF-HFP	Fluorinated backbone; HFP lowers crystallinity	Good chemical and electrochemical stability; strong film formation; high electrolyte uptake in porous forms	Crystalline PVDF can restrict ion transport; fluoropolymer processing often requires organic solvents
PEO	Ether-oxygen-rich flexible chain	Strong Li-salt solvation; flexible chemistry; well-understood transport mechanism	Crystallinity and limited oxidative stability can constrain room-temperature/high-voltage performance
PAN	Polar nitrile groups	Good solvent compatibility, thermal stability, and potential for high conductivity	Interfacial compatibility and mechanical behavior depend strongly on composition and support structure
PMMA	Carbonyl-rich side groups; readily crosslinked	High affinity for carbonate solvents; tunable network density and mechanical properties	Mechanical strength and electrolyte retention must be balanced with crosslink density
Cellulose-derived matrices	Renewable polysaccharide framework with hydroxyl functionality	High mechanical strength, sustainability, chemical modifiability	Moisture sensitivity and process control are important for battery-grade use

**Table 2:** Representative GPE systems and reported cell-level performance. Values should be compared with caution because electrode loading, electrolyte quantity, voltage window, temperature, and cell format differ among studies.

GPE Design	Cell Chemistry	Voltage/Condition	Reported Performance	Purpose	Source
PVDF-HFP + ionic liquid	Li|GPE|LFP	Stability to ~5.5 V; 20 °C	~160 mAh g^−1^ in the reported Li/LFP cell	High conductivity and electrochemical stability	[30]
PVDF/HEC/PVDF sandwich	Li|GPE|LFP	0.2 C	~140 mAh g^−1^ over ~140 cycles	Mechanical support and anion interaction	[40]
PVDF-HFP + PEGDME	Li|GPE|LFP	C/10 to moderate rates	~98% retention after 60 cycles; nonflammable GPE	Safety and flexible processing	[51]
PVDF-HFP/PEO + PEG-UPy	Li|GPE|LFP	1 C	~163 mAh g^−1^; stable operation over 200 cycles	Room-temperature self-healing	[24]
PVDF-HFP/PEO/SiO2	Li|GPE|LFP	Long-cycle test	~147.7 mAh g^−1^ after 200 cycles	Porosity, uptake, and mechanical reinforcement	[50]
Fluorinated PEGDA GPE	Li|GPE|LCO	−40 °C to room temperature; up to ~5.6 V stability	~92.9% capacity retention after 400 cycles at 1 C	Wide-temperature operation and high-voltage stability	[69]
PVDF-HFP/LAGP/PVAc/LiTFSI composite	Li|GPE|LFP	2 C, 25 °C	~97.6% capacity retention after 500 cycles	Rigid-flexible network and stable interface	[74]
Flame-retardant polyacrylate GPE	Li|GPE|NCM811	3.0–4.7 V, 2 C	~82.2% retention after 100 cycles	Noncombustibility and high-voltage safety	[76]
Crosslinked self-healing GPE	Li|GPE|NCM811	2.8–4.7 V, 0.5 C	Up to 500 cycles in the reported system	Self-healing and high-voltage Ni-rich cathode compatibility	[89]
3D crosslinked c-GPE-50	Li|GPE|NCM622	0.5 C	~160 mAh g^−1^; ~80% after 300 cycles and ~74% after 400 cycles	High-voltage stability and crack mitigation	[71]
PVDF-HFP GPE + TMSPB additive	Li|GPE|NCM811	3.0–4.35 V	~94% retention in the reported comparison; 133.5 mAh g^−1^ at 15 C	Stable Ni-rich cathode/electrolyte interface	[77]
In situ polymerized GPE	Si/C@C-Gr|GPE|NMC pouch cell	2.7 Ah pouch format	325.9 Wh kg^−1^ and 88.7% capacity retention over 2000 cycles	Practical full-cell interface stabilization	[90]
3D interpenetrating nonflammable GPE	LFP|GPE|graphite and related cells	1 C; safety tests	Stable cycling and 63.3% reduction in maximum surface temperature during reported safety test	Nonflammability and practical LIB safety	[91]
CF_3_-/N-H functional in situ GPE	Graphite|GPE|NCM811	3 C (1.8 mA cm^−2^)	73.2% capacity retention after 300 cycles; 2.6 mS cm^−1^; tLi^+^ = 0.67	Fast Li^+^ transport and anion anchoring	[86]
PEGDMA/DFEA LiF-forming GPE	NCM811|GPE|graphite pouch cell	4.38 V; 45 °C	250 Wh kg^−1^; 94.0% retention after 400 cycles at 45 °C	LiF-rich interfaces, high-temperature cycling, abuse safety	[87]
EC-free in situ quasi-solid electrolyte	High-Ni NCM|GPE|graphite Si Ah pouch cell	Hot-box/thermal-abuse tests	No thermal runaway until 180 °C in reported hot-box test; electrochemical performance maintained	Practical thermal safety via in situ polymer network	[88]
Multiple H-bond self-healing GPE	SiOx/graphite|GPE|NCM811 Ah pouch cell	Pressure-free cycling	1000 cycles at 0.5 C (0.8 A) in the reported Ah-scale pouch cell	Adaptive interface mechanics and swelling suppression	[57]
EC-less low-reactivity in situ GPE	NCM811|GPE|graphite pouch cell	1 Ah; thermal-runaway evaluation	Peak temperature reduced to 484 °C from 547.2 °C for liquid-electrolyte control; comparable cycling	Reduced heat release, gas generation, and electrode crosstalk	[79]

**Table 3:** Major challenges in GPE development and practical design responses.

Challenge	Underlying Cause	Promising Design Response	Key Validation
**Conductivity vs. mechanical strength**	High liquid content and chain mobility improve transport but soften the matrix	Dual networks, moderate crosslinking, reinforced porous frameworks	Conductivity + tensile/mechanical testing at the same formulation
**High-voltage instability**	Oxidation of solvent/polymer and unstable cathode interphase	Fluorinated chemistry, reactive additives, cathode-compatible interphase design	LSV/CV plus long-term high-voltage cycling and interface analysis
**Interfacial resistance**	Poor wetting/contact or continuous side reactions	In situ polymerization, adhesive networks, surface-active functional groups	EIS evolution, cross-sectional morphology, full-cell cycling
**Wide-temperature operation**	Low-temperature mobility loss; high-temperature softening and side reactions	Low-volatility plasticizers, flexible networks, thermally robust crosslinks	Conductivity and cell tests across the claimed temperature range
**Safety and flammability**	Retained organic liquid may remain combustible	Ionic liquids, flame-retardant additives, low-volatility phases, nonflammable networks	Ignition/self-extinguishing tests, thermal shrinkage, abuse tests
**Scale-up and reproducibility**	Multicomponent formulations and laboratory-specific processing	Simplified chemistries, solvent-efficient processing, controllable in situ curing	Large-area membrane uniformity, pouch-cell validation, process repeatability
**Practical-cell validation**	Half-cell and low-loading coin-cell tests do not capture electrolyte inventory, electrode balancing, gas generation, or cell-level thermal behavior	Graphite/Si full cells, realistic electrode loading, controlled N/P ratio, scalable in situ processing	Ah-scale pouch-cell cycling, energy density, storage, fast-charge and abuse tests

## Data Availability

No new data were created or analyzed in this study. Data sharing is not applicable to this article.
